# Non-hypothetical projection pursuit regression for the prediction of hydration heat of Portland-cement-based cementitious system

**DOI:** 10.1016/j.heliyon.2023.e19471

**Published:** 2023-08-28

**Authors:** Can Qin, Jingwei Gong, Gangchuan Xie, Jianxin He, Liang Liu, Haihua Yang, Chuanling Deng

**Affiliations:** a College of Hydraulic and Civil Engineering, Xinjiang Agricultural University, Urumqi, Xinjiang, 830052, PR China; b Xinjiang Key Laboratory of Hydraulic Engineering Security and Water Disasters Prevention, Urumqi, 830052, PR China; c College of Mechanical and Electrical Engineering, Xinjiang Agricultural University, Urumqi, Xinjiang, 830052, PR China

**Keywords:** Hydration heat, Non-assumptive projection pursuit regression, Portland cement based cementitious system, Ridge function, Iterative optimization method

## Abstract

In this study, the non-hypothetical projection pursuit regression (NH-PPR) is proposed. The proposed NH-PPR model can predict the hydration heat based on the four cement phases, FA, SL, cement fineness and hydration time. The NH-PPR model is proposed by using the multiple layer iteration method and the non-hypothetical and non-parametric ridge functions to enhance accuracy and solve the problems caused by the parameter selection and the subjective hypothesis. The modeling data set is applied to train model, the testing data set is regressed and fitted into the model, and then the obtained results are compared with the BP model. To further validate the proposed model, another published data set is used to obtain a higher degree of confidence in the prediction. It is shown that the proposed model obtains the better accuracy, stability and versatility, and avoids the parameter selection and subjective hypothesis.

## 1 Introduction

Portland cement has been widely used in infrastructure construction [1]. It is worth noting that cracks often appear in concrete, which can affect the durability and service life of infrastructure construction [2,3]. Thermal gradients are especially problematic in mass concrete constructions, where high thermal stresses can increase the early cracking [[4], [5], [6]]. With limited thermal conductivity of concrete, the heat generated by hydration of cement is difficult to release, resulting in significant thermal gradients and thermal stresses [[7], [8], [9]]. Therefore, thermal cracking of concrete is mainly influenced by the hydration heat of cementitious materials.

Studies on the hydration heat of cementitious materials are usually measured experimentally. However, conventional test experiments are time-consuming, laborious and costly, especially for multiple cementitious materials [10]. Therefore, it is necessary to propose predictive models to avoid experiments. On the one hand, early work to develop hydration heat models focused on the contributions of the cement phases (C_3_S, C_2_S, C_3_A, and C_4_AF), cement compositions, and cement fineness (CF) [11]. Woods et al. presented the hydration heat equations using a linear regression for the four major phases based on the experimental data of 13 cements [12,13]. The results indicated that there are good linear correlations between the final hydration heat and the amount of C_3_S+2.1C_3_A. Verbeck et al. implemented a least-squares fit of the experimental data hypothesizing linear and independent relationships between the four major cement phases, SO_3_ and hydration time, and proposed the hydration heat equations [14]. Poole and Taylor developed the hydration heat equations to predict the seven-day hydration heat as a linear function for the four major cement phases and CF based on the data retrieved from the report of Verbeck et al. [[14], [15], [16]].

On the other hand, extensive research has shown that the hydration heat can be significantly reduced with partial replacement of cement by supplementary cementitious materials (i.e., slag and fly ash) [10,11,16,17]. A number of models have been proposed to predict the hydration heat released from the mixed cements as well as the binary combinations of ordinary Portland cement and slag (SL) [[18], [19], [20], [21], [22]]. Wang et al. proposed a exothermic equation to calculate the hydration heat of mixtures containing SL and fly ash (FA) [23]. Schindler and Folliard built a hydration heat model, which contains the effects of SL and FA [24,25]. Poole and Riding et al. demonstrated that the chemical admixtures and the cement fineness (CF) would affect the hydration process [26,27].

It is clear that the above hydration heat models were developed by fitting experimental data using linear regression method based on the subjective hypothesis of a linear (or nonlinear) independent relationship between hydration heat and influencing factors. These modeling methods are considered as confirmatory data analysis (CDA) methods. Notably, the accuracy of these modeling methods depends on whether the subjective hypotheses are consistent with objective reality. Besides, these modeling methods are mostly empirical leading to the prediction of hydration heat is often limited by a certain range of input variables. Therefore, there is an urgent necessity for an excellent and objective method to predict the hydration heat.

In recent years, there has been an increasing interest in Exploratory data analysis (EDA) methods, which are based on the fundamental principles of experimental data and avoids subjective hypotheses. Although the concept of EDA was defined decades ago, powerful modeling tools for EDA have emerged with the development of machine learning and data mining algorithms. Recently, among the different EDA methods, intelligent computational methods, such as evolutionary computation and artificial neural networks (ANNs), have been widely applied in cement or concrete related modeling studies with remarkable results. The structure of neural network is composed of an input layer, a hidden layer and an output layer, and the connections between neurons from one layer to another are programmed [28]. Subasi et al. and Binici et al. introduced adaptive neuro-fuzzy inference systems (ANFISs) and genetic expression programming (GEP) for the prediction of the early hydration heat of plain and blended cements [29,30]. Park et al. proposed prediction approaches for hydration reactions using backpropagation (BP) neural networks [31]. Trtnik et al. established a predictive model for the adiabatic temperature rise during concrete hydration with the use of artificial neural networks (ANN) and attained good prediction accuracy [32]. Wang et al. modeled the early-age hydration kinetics and the evolution of the hydration reactions of Portland cement using the flexible neural tree and the feedforward neural network (FNN) [33,34]. Ozbay et al. introduced genetic programming (GP) as a tool to study the fresh and hardened properties of self-compacting concretes with various formulations [35]. Despite their many advantages, neural network models have several drawbacks. such as over-fitting problems, due to the non-selective chosen neural networks with many hidden layers and/or hidden neurons in each layer [36,37]. Besides, these models need to identify many parameters that are not easily available to depict the complicated connections between the hydration heat and its influencing factors. Therefore, it is important to apply EDA methods with few subjective parameters to predict the variation of the hydration heat with the influencing factors.

Recently, among different EDA methods, projection pursuit regression (PPR) method has widely been used in many fields, such as the analysis of industrial data, the assessment of crop protection, the prediction of corrosion in metallic materials and the prediction of hydrology [[38], [39], [40], [41], [42], [43], [44]]. The traditional PPR method not only hypothesizes that the ridge function is the given hermite function and the polynomial function, but also employs the multiple-objective genetic algorithm and the coarse-grained genetic algorithm to select parameter [40,42]. It is worth noting that the parameter selection and subjective hypotheses would lead to the non-uniqueness problem of computed values.

In this study, the non-hypothetical PPR (NH-PPR) method to predict the hydration heat for Portland-cement-based cementitious systems was proposed. The non-hypothetical and non-parametric ridge function in the form of numeric function (data table) and the multiple layer iteration method were applied to improve the NH-PPR method, which improved the accuracy of model and solve the problem of non-uniqueness of computed values caused by parameter selection and subjective hypotheses. Data set 1 (experimental data) and data set 2A (previously reported data) were divided into modeling and testing data sets. Training the model using the modeling data set, regressing and fitting the model using the testing data set, and then the obtained results were compared with the linear regression models and the Levenberg-Marquardt-type back-propagation (BP) artificial neural network. In order to further verify the presented model, another data set was utilized to attain higher degree of verification.

## 2 NH-PPR model

### 2.1 Conventional PPR model

To explain the calculation steps and basic theory, the PPR model is depicted as

(1) $$G\left(U\right)=E\left(V_{n}|U_{1}\ldots U_{M}\right)=EV_{n}+\sum_{k=1}^{K}\mu_{k}\cdot g_{k}\left(\sum_{m=1}^{M}\eta_{mk}\cdot u_{m}\right)$$

where *U* is the independent variable, *V* is the dependent variable, *G*(*U*) is the projection pursuit regression, *k* is the subfunction approximation index, *K* is the optimal number of ridge functions, *g*_*k*_ is the *k*th ridge function, $\eta_{mk}$ is the *k*th linear coefficient and $\mu_{k}$ is the weight coefficient of the *k*th ridge function.

The *M*_2_ minimization criteria is expressed as

(2) $$M_{2}=\sum_{n=1}^{N}W_{n}E{\left[V_{n}-EV_{n}-\sum_{k=1}^{K}\mu_{k}\cdot g_{k}\left(\sum_{m=1}^{M}\eta_{mk}\cdot u_{m}\right)\right]}^{2}$$

where *W*_*n*_ is the weight coefficient.

### 2.2 The development of NH-PPR model

**Fig. 1:**
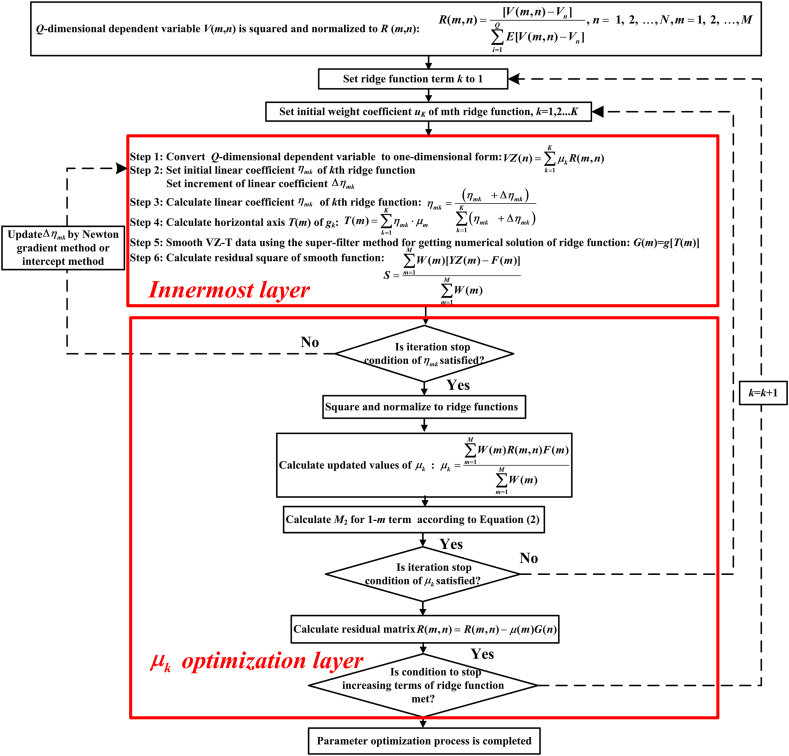
Flowchart of parameter optimization process.

Firstly, the multiple layer iteration method was adopted to the NH-PPR model. The *M*-dimensional independent variables *U* were squared and normalized so that *U* satisfied the *M*_2_ minimization criterion, and then $\mu_{k}$, *g*_*k*_ and $\eta_{mk}$ were optimized in three layer grouping. Notably, the innermost layer is used to optimize *g*_*k*_ and $\eta_{mk}$. Therefore, the non-hypothetical and non-parametric function associated the dependent variables and the independent variables. The specific optimization process is depicted in Fig. 1 and Appendix B in Supplementary Materials.

It is clear from Fig. 1 that the numerical functions (data tables) were used in NH-PPR model to present the non-hypothetical and non-parametric ridge functions to avoid the problems of hypothesizing the ridge functions form and the disadvantages of subjective calculation results. Therefore, one can conclude that the problems of subjective hypotheses and parameter selection that exist in CDA methods are thus avoided.

## 3 Data collection

### 3.1 Data set 1: experimental data

#### 3.1.1 Materials and mixture proportions

There are seven Portland cements with two mineral admixtures (FA and SL) selected for the study, which are widely adopted for mass structural concrete. Portland cements consisted of two types of low-heat cement (L1 and L2), two types of ordinary Portland cement (O1 and O2), one type of moderate-heat cement (M1), and two types of high sulfate resistant Portland cement (H1 and H2). The chemical components of cement phases, FA and SL are listed in Table 1. The physical properties and the mineral composition are listed in Table 2.

**Table 1:** Chemical components of Portland cement, SL and FA.

Analysis (*wt*%)	SiO_2_	Al_2_O_3_	Fe_2_O_3_	CaO	MgO	SO_3_	*f*-CaO
L1	23.0	4.6	5.5	61.4	1.3	0.8	0.3
L2	23.5	4.3	4.8	61.6	1.7	2.3	0.5
O1	21.8	4.6	3.6	66.7	1.2	0.8	1.6
O2	22.0	4.7	3.3	66.9	1.7	0.3	0.9
H1	22.4	4.5	5.6	64.1	0.8	1.9	0.5
H2	23.9	4.4	6.1	62.8	1.2	0.6	0.4
M1	22.6	4.8	4.1	64.1	1.0	0.8	1.1
FA	52.4	20.9	7.1	7.7	2.9	1.0	–
SL	34.4	11.2	1.2	41.0	5.8	0.7	–

Note: L: low-heat Portland cement, O: ordinary Portland cement, H: high-sulfate-resistant Portland cement, M: moderate-heat Portland cement, FA: fly ash, SL: slag.

**Table 2:** Mineral composition and physical properties of Portland cements, SL and FA.

Physical properties		L1	L2	O1	O2	H1	H2	M1	FA	SL
Speciﬁc gravity/g·m^−3^		3.2	3.2	3.2	3.2	3.2	3.2	3.2	2.4	2.9
Cement fineness/m^2^·kg^−1^		320	316	350	358	364	342	331	383	439
Normal consistency/%		26.6	26.4	28.0	27.2	26.3	27.1	27.4	–	–
Initial setting time/min		187	129	166	220	173	168	151	–	–
Final setting time/min		241	220	220	265	228	224	230	–	–
Activity index/%	7 d	–	–	–	–	–	–	–	69	57
	28 d	–	–	–	–	–	–	–	83	91
Mineral composition/%	C_3_S	34.2	30.1	65.4	64.2	37.8	32.3	34.4	–	–
	C_2_S	40.4	44.8	13.9	15.8	35.6	44.6	39	–	–
	C_3_A	2.8	3.2	6.8	6.1	0.1	1.4	1.8	–	–
	C_4_AF	16.6	14.7	10.0	10.8	18.5	18.4	34.4	–	–

According to the previous studies, the content of four cement phases (C_3_S, C_2_S, C_3_A, and C_4_AF), CF, FA, SL, and hydration time were chosen as influencing factors. The mortar mixture design and sample names are summarized in the **Appendix** Table A1 of Supplementary Material [29,45,46].

#### 3.1.2 Test procedure

The seven-day hydration heat for cement and mortar according on GB/T 12959-2008 was carried out by the direct method, which is basically consistent with the semi-adiabatic calorimetry method of EN 196-9 [[47], [48], [49]]. For the direct method, 450g cementitious material and 1350g ISO standard sand was mixed as mixture. The normal consistency of cementitious added 5% is the water requirement of mixture. About 800g mixtures were placed in glass jar, sealed and placed in a constant temperature water pool at 20 °C. Notably, the water requirements and external temperature have been determined in this experiment. Here, this study ignored the water and temperature on the experiment. The seven-day hydration heats were determined by directly measuring the temperature change and calculating the total heat loss [48,50].

### 3.2 Data set 2: published data

Two data sets from the published literature were considered in this study. The first was data set 2A, which consisted of 172 test results. The materials contained high proportions of mineral admixtures and 31 types of cements, including the second type of moderate-heat cement (M2) [[51], [52], [53]]. The ranges of the C_3_S, C_2_S, C_3_A, C_4_AF, FA, SL, CF and hydration time were 22%–74.7%, 2.4%–54.6%, 0.1%–13.2%, 6.4%–16.7%, 0%–70%, 0%–70%, 285–489 m^2^/kg, and 1–7 days, respectively. The second was the data set 2B, which consisted of three mixtures (21 samples), provided by Li et al. [54] The cement used in the experiment was the third type of moderate-heat cement (M3). Notably, the eight dependent variables of published data set 2B were within the modeling data set (data sets 1 and 2A). The model was developed by data sets 1 and 2A, which was hypothesized to be capable of predicting the hydration heat accurately, and then the model was validated by calculating the data set 2B. Some of the published data did not provide the four phases of cement, which were calculated by the Bogue equation [55]. Data sets 2A and 2B are shown in Appendix Table A2 and Table A3 of Supplementary Material, respectively.

**Table 3:** Comparison of the modeling and testing data set results.

Data set	Qualification rate	*R*	RMSE	MAE	RRMSE	RMAE
	%		(J/g)	(J/g)	(%)	(%)
Modeling data set	75.2	0.980	11.8	8.3	5.9	4.1
Testing data set	73.6	0.969	13.7	8.9	7.3	4.7

Note: RMSE: root mean square error, MAE: mean absolute error, RRMSE: relative RMSE, RMAE: relative MAE.

### 3.3 Performance evaluation methods

The performance indicators were used to evaluate the accuracy of proposed model. These indicators are defined as follows [56,57].

(3-1) $$R=\frac{\sum_{i=1}^{n}\left(y_{i}-\bar{y}\right)\left(y_{i}^{pre}-{\bar{y}}^{pre}\right)}{\sqrt{\sum_{i=1}^{n}{\left(y_{i}-\bar{y}\right)}^{2}\sum_{i=1}^{n}{\left(y_{i}^{pre}-{\bar{y}}^{pre}\right)}^{2}}}$$

(3-2) $$RMSE=\sqrt{\frac{1}{n}\sum_{i=1}^{n}{\left(y_{i}-y_{i}^{pre}\right)}^{2}}$$

(3-3) $$MAE=\frac{1}{n}\sum_{i=1}^{n}\left|y_{i}-y_{i}^{pre}\right|$$

(3–4) $$RRMSE=\frac{RMSE}{\bar{y}}\times 100$$

(3–5) $$RMAE=\frac{MAE}{\bar{y}}\times 100$$

where *n* is the number of data, and $\bar{y}$ and ${\bar{y}}^{pre}$ are the actual and predicted mean values, respectively. When correlation coefficient *R* is close to one, the actual and predicted values vary similarly. The MAE measures the average magnitude of the errors between the actual and predicted values, and the RMSE measures the average magnitude of the error. However, *R*, RMSE, and MAE are insufficient for evaluating the accuracy of the model, because they do not change significantly when $y_{i}^{pre}$ is multiplied by a constant. The percentage deviations of the actual and predicted data were investigated using the RRMSE and RMAE. In summary, the established model has a higher accuracy, with higher the *R* value, lower the RMSE, MAE, RRMSE and RMAE values, and lower the errors between the actual and predicted values.

## 4 Results and discussion

### 4.1 Calculation the hydration heat using NH-PPR model

#### 4.1.1 Parameter setting

To build and validate the model, samples from data sets 1 and 2A were randomly split into the modeling (80%) and testing (20%) data set, respectively. The modeling data set was used to build the model, and the testing data set was then regressed and fitted into the model to validate the performance. Although the established model has high accuracy for the calculating results of modeling data set, the predictive performance of the testing data set can not be guaranteed, which means the stability performance of established model may be poor. Therefore, this study proposed a “consistency test” criterion to ensure the stability of the NA-PPR model, which is that the fitting precision of the modeling data set is consistent with the predicted precision of the testing data set using performance evaluation methods.

To improve the stability and accuracy of the predictive models in this study, it was necessary to expand the range of input variables. Therefore, based on the sample selection criteria, data sets 1 and 2A (550 samples) were divided into two data sets: 444 samples (modeling data set) and 106 samples (testing data set), which consisted of one dependent variable (*Y*) and eight independent variables for C_3_S (*x*_1_), C_2_S (*x*_2_), C_3_A (*x*_3_), C_4_AF (*x*_4_), FA (*x*_5_), SL (*x*_6_), CF (*x*_7_) and hydration time (*x*_8_).

According to the modeling data set, a series of parameters were set: *M* = 8, *K* = 3, *S* = 0.3, N = 440, and *Q* = 1, where *M* is the number of input variables, *Q* is the number of output variables, *N* is the number of modeling samples, *K* is the optimal number of ridge functions, respectively. Besides, *S* determines the model fineness when analyzing the internal structure of the data. It is worth noting that *S* is the only one parameter for NH-PPR model that is required to be chosen by “consistency criterion”. The problem of model uncertainty caused by subjective hypotheses and artificial parameter assignment was avoided because the parameters values of the NH-PPR modeling were objective and verifiable. That is the reason why the model is described as the non-hypothetical PPR model.

#### 4.1.2 Information mining

The linear coefficients were depicted as follows:

(4) $$\left(\begin{array}{c} \eta_{1}\\ \eta_{2}\\ \eta_{3} \end{array}\right)=\left(\begin{array}{cccccccc} -0.0534 & -0.2068 & 0.3938 & 0.2409 & -0.1264 & -0.1045 & 0.0323 & 0.8446\\ 0.0209 & -0.0487 & 0.0587 & -0.0070 & 0.0061 & 0.0047 & -0.0068 & 0.9942\\ -0.0384 & 0.2845 & -0.2673 & -0.8778 & 0.0097 & 0.0429 & 0.0031 & -0.2713 \end{array}\right)$$

The contribution weight coefficients were depicted as follows:

(5) μ=(0.8425,0.3677,0.1896)

**Fig. 2:**
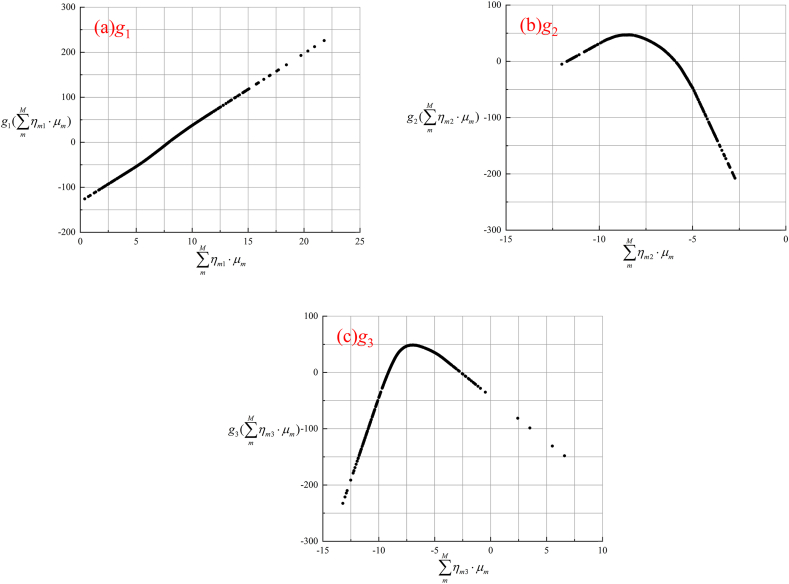
Ridge functions of the NH-PPR model for the data sets 1 and 2A: (a)*g*_1_, (b)*g*_2_, (c)*g*_3_.

To make the ridge function more intuitive and clear, the numeric function (data table) would be represented by a scatter plot, as depicted in Fig. 2(a–c). Besides, the data table form is presented in Table A4 of Supplementary Material. Finally, the NH-PPR model is obtained by introducing Equation (1), Equation (4), and Equation (5) and Table A4 into Equation (2).

**Table 4:** Parameters of the linear regression models.

Model	*a*	*b*	*c*	*d*	*e*	*f*
Taylor [16]	1.53	0	11.44	0	0.4	1.98
Poole-1 [15]	2.22	0.42	15.57	4.94	0	0
Poole-2 [15]	2.13	0	9.36	0	0	133.9

#### 4.1.3 Accuracy analysis

In order to further demonstrate the performance of the constructed hydration heat model, the qualiﬁcation rate was proposed. The qualiﬁcation rate was the percentage of qualiﬁed samples relative to the total number of samples. When the relative error between the actual and predicted values of the sample was less than 6%, the sample was considered qualiﬁed. The relative error was set to 6% because the deviation of the test error and the average value of the hydration heat on the seventh day were 12 J/g and 200 J/g, respectively.

**Fig. 3:**
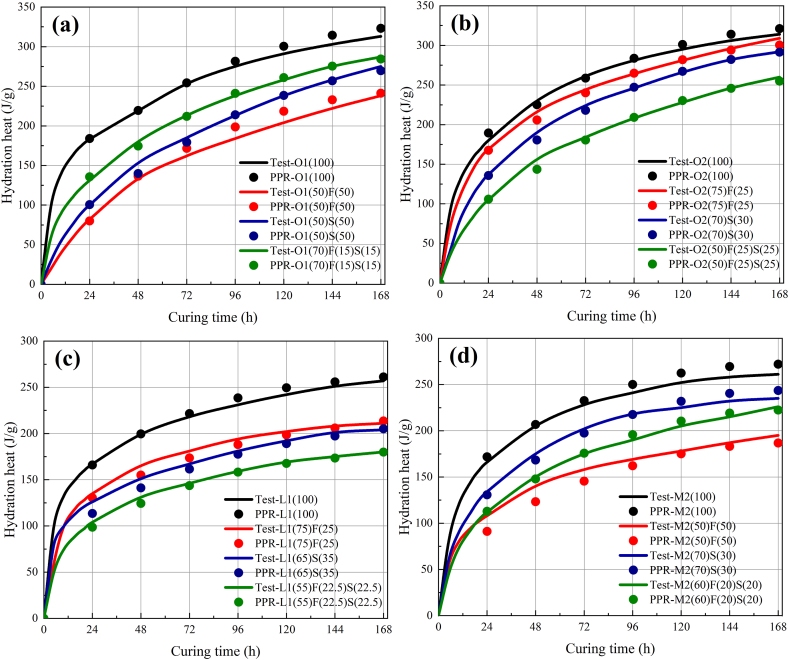
Calculation results of the 1- to 7-day hydration heats for data sets 1 and 2A: (a), (b), (c) test results from experiments, and (d) test results according to the Wu et al. (2005). (a) ordinary Portland cement 1; (b) ordinary Portland cement 2; (c) low-heat Portland cement 1; (d) moderate-heat Portland cement 2.

**Fig. 4:**
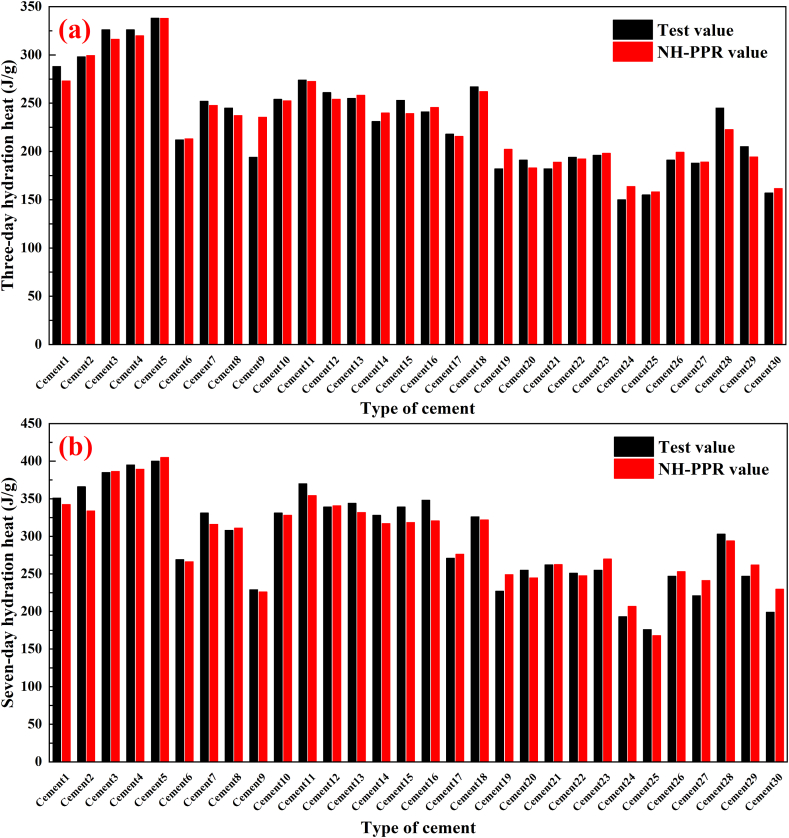
Comparison of results calculated using the NH-PPR model for data set 2A. (a) Three-day hydration heat; (b) Seven-day hydration heat.

**Fig. 5:**
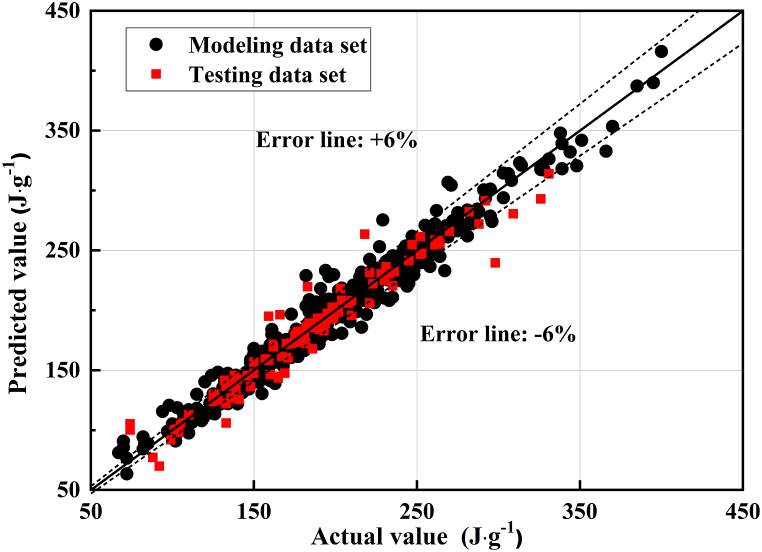
The experimental and predicted values of the data sets 1 and 2A using the NH-PPR model.

The comparison between modeling and testing data set of the NH-PPR model for the data sets 1 and 2A are given in Table 3. The performance indicators used to evaluate the accuracy of model are calculated from Equation (3). The NH-PPR model predicted the modeling and testing data sets well, as depicted in Table 3, with qualification rates of 75.2% and 73.6%, RMSEs of 11.8 and 13.7 J/g, and MAEs of 8.3 and 8.9 J/g, respectively. The NH-PPR model calculations values are illustrated in Fig. 3(a–d) and Fig. 4(a–b). Fig. 5 depicts the experimental and predicted values of data sets 1 and 2A.

One can conclude that the NH-PPR model predicted values were close to the experimental values. It should be emphasized from Fig. 3(a–d) that the calculation results for the hydration heat at different hydration times (three and seven days) had reasonably good predictions. Besides, the differences of the modeling and testing data set in qualification rates and root mean square errors (RMSEs) are 1.6% and 1.9 J/g in Table 3. The obtained results for the data sets 1 and 2A have revealed that the NH-PPR model conforms to the “consistency criterion” and has high stability. Therefore, one can conclude that the presented NH-PPR model provided a trustworthy prediction tool for the hydration heat for engineering applications.

### 4.2 Comparative study

The presented NH-PPR has pleasant performance in predicting the hydration heat as depicted in the previous section. The linear regression model and the artificial neural network was used for the calculation and analysis of the modeling and test data sets to further demonstrate the merits of the NH-PPR model.

#### 4.2.1 Compared with linear regression models

**Fig. 6:**
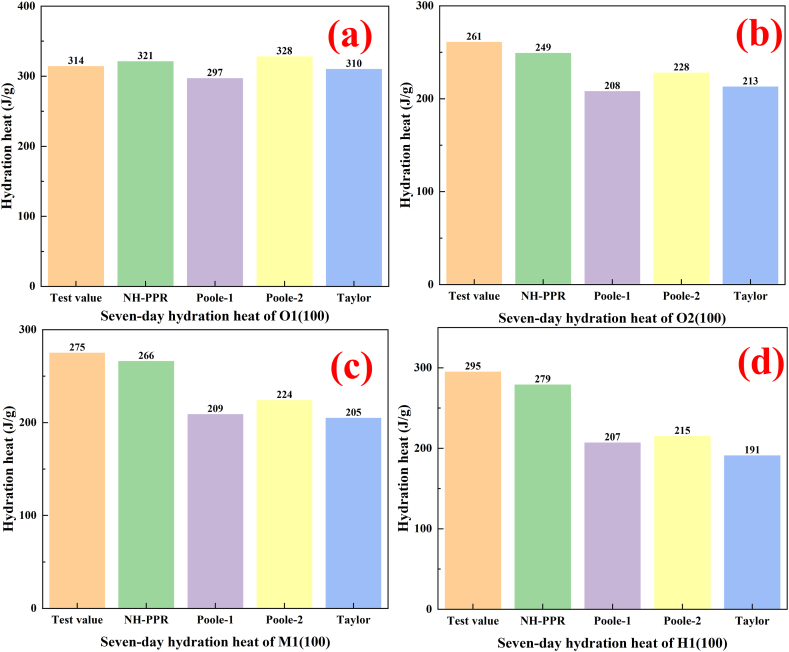
Comparison of results calculated using the linear regression model for data set 1.(a) ordinary Portland cement 2; (b) low-heat Portland cement 2; (c) moderate-heat Portland cement 1; (d) high-sulfate-resistant Portland cement 1.

Taylor and Poole proposed the linear regression models for predicting the seven-day hydration heat of cement were summarized as follow [15,16].

(6) $$Q\left(t\right)=a\cdot P\left(C_{3}S\right)+b\cdot P\left(C_{2}S\right)+c\cdot P\left(C_{3}A\right)+d\cdot P\left(C_{4}AF\right)+e\cdot CF+f$$

where *P*(*C*_3_*S*), *P*(*C*_2_*S*), *P*(*C*_3_*A*) and *P*(*C*_4_*AF*) is the mineral content of cement, %; *Q*(*t*) is the hydration heat of the cement, J/g, *CF* is the cement fineness, m^2^/kg. Table 4 shows the parameters of the linear regression models in Equation (6). Fig. 6(a–d) demonstrated a comparison between predictive results of seven-day hydration heat of cement with experimental observation.

It is shown from Fig. 6(a–d) that the Poole-2 model was better than the Poole-1 model and Taylor model in the prediction of seven-day hydration heat of cement. The Poole-2 model produced the smallest MAE (44.5 J/g) in the linear regression models for the four cements. However, the NH-PPR model offered the highest level of prediction accuracy with the smallest MAE (11 J/g). Besides, Taylor, Poole-1 and Poole-2 model is only suitable for O2_(100)_ cement (MAE for the was 4 J/g, 17 J/g and 14 J/g, respectively). Compared to the linear regression models, one can conclude that the NH-PPR model has a higher accuracy and can be applied to various Portland cements. This can be attributed to NH-PPR model avoided the subjective hypotheses in the linear regression models, which hypothesized the linear and independent relationships between the four mineral compositions and the hydration heat. Moreover, the parameters of the linear regression model were obtained by regression fitting the experimental data of ordinary Portland cement, resulting in inapplicability to other Portland cements.

#### 4.2.2 Compared with BP model

The most widely used and effective artificial neural network with the Levenberg-Marquardt-type back-propagation (BP) algorithm was chosen for comparison [[58], [59], [60], [61]]. The structure of proposed BP neural network consisted of an input layer containing eight neurons representing the influence factors (i.e., hydration time, CF, and contents of FA, SL, C_3_S, C_2_S, C_3_A, and C_4_AF), the hidden layer, and an output layer containing one neuron, which output the hydration heat. To avoid the effect of randomness of data, the BP model adopted the same data set as the NH-PPR model. In this study, the modeling process is completed when the learning cycle is equal to 1000. Notably, in the process of selecting architecture, it was found that the BP model calculation results for the same data set would be different if the architecture settings were different. By compared the prediction results of several calculations, the appropriate architecture (i.e., one hidden layer, 30 neurons, and 0.035 learning rate) for the BP model was decided.

**Fig. 7:**
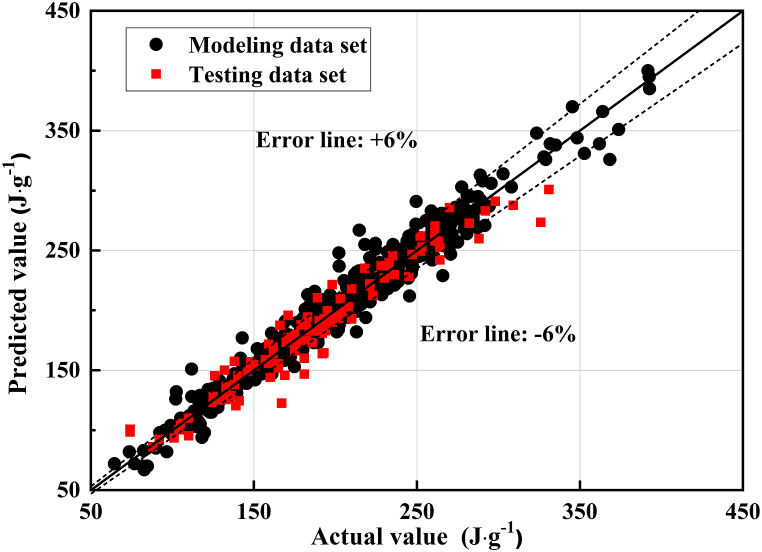
The actual and predicted hydration heats of data sets 1 and 2A using the BP model.

After the architecture of the BP model was determined, the calculated result of the BP model was compared with NH-PPR model. Fig. 7 shows the predicted hydration heats of data sets 1 and 2A using the BP model. As depicted in Fig. 7 that the RMSE and MAE of the predicted low-hydration-heat (＜250J/g) value were 13.6 J/g and 9.4 J/g, and the RMSE and MAE of the predicted high-hydration-heat (＞250J/g) value were 14.2 J/g and 9.8 J/g. The proposed BP model could provide good predictions for the low-hydration-heat samples but not high-hydration-heat samples. A comparison of the calculation results have demonstrated that the NH-PPR model outperformed the BP model in terms of prediction accuracy and stability for data sets 1 and 2A.

**Fig. 8:**
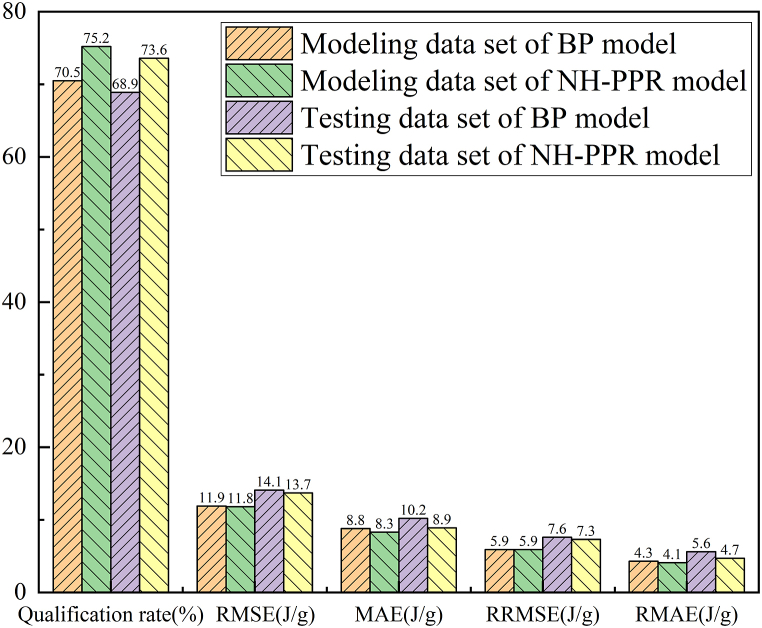
Comparing the Calculation result of the BP model with NH-PPR model.

As shown in Fig. 8, the difference of the modeling and testing data set between the qualification rate, RMSE, and MAE were 1.6%, 2.2 J/g and 1.4 J/g for the BP model, respectively. The results also indicated that the BP model also met the consistency criterion. As shown in Fig. 8, the qualification rate of the BP model was approximately 70%, but the qualification rate of NH-PPR model was 74%. In addition, the RMSE and MAE of the BP model were higher than those of the NH-PPR model.

Based on the above comparison results, it can be seen that the BP model requires many subjective assignments for multiple parameters (i.e., the number of hidden layers, the number of neurons in the hidden layer, and the learning rate), leading to a certain subjectivity in the BP model. Moreover, these parameters lack an objective and accurate selection criterion, resulting in the non-uniqueness problem of calculation results for the BP model. Contrarily, only *S* parameter is required to be chosen by the “consistency criterion” in the NH-PPR model. Therefore, the proposed NH-PPR model has excellent objectivity and is a prospective non-hypothetical EDA method.

### 4.3 Calculation for new data set

The proposed NH-PPR model was verified with experimental data (data set 1) and published data (data set 2A) in the previous sections. The NH-PPR has demonstrated excellent predictive performance for hydration heat. To show that the NH-PPR model can help researchers and engineers solve prediction problems, the presented NH-PPR model was then verified with new data set 2B, as described in this section.

**Fig. 9:**
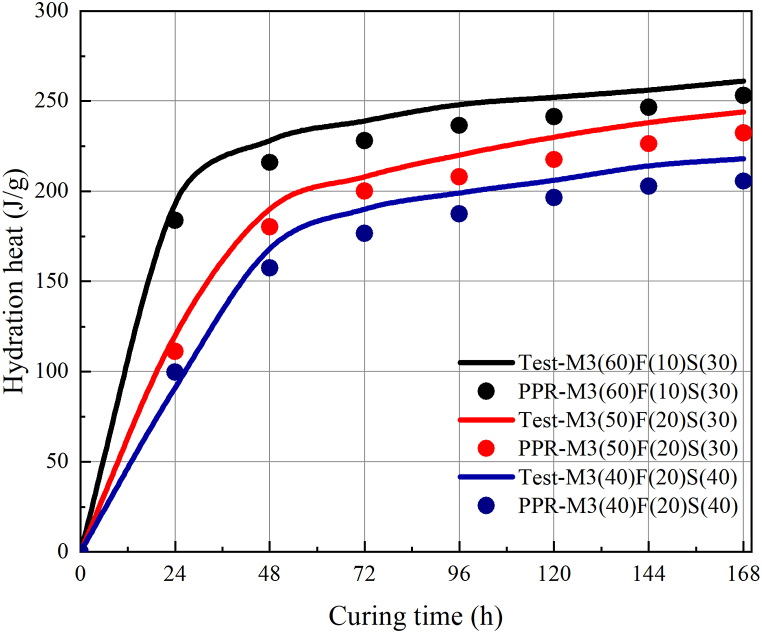
The calculated results of the NH-PPR model for data set 2B.

As shown in Fig. 9, the hydration heat for data set 2B was calculated by the NH-PPR model. Although data set 2B contained new data for the trained model, the model was not guaranteed to obtain accurate predicted values. However, the NH-PPR model can accurately predict the hydration heat, and the 10.2 J/g for RMSE and the 9.8 J/g for MAE are both acceptable. From Fig. 9, it is observed that the calculated values were consistent with the experimental values. According to data sets 1, 2A, and 2B, the NH-PPR model has provided an efficient tool to evaluate the hydration heat.

## 5 Conclusions

In this study, a new non-hypothetical projection pursuit regression (NH-PPR) was presented. The developed NH-PPR model can predict the hydration heat based on C_3_S, C_2_S, C_3_A, C_4_AF, FA, SL, CF, and hydration time. Three data sets from experiments and the literature were used to train and validate the NH-PPR model. Compared with the linear regression models and the BP neural network model, the NH-PPR model had higher accuracy, stability, and versatility. Furthermore, NH-PPR model was proposed by using the non-hypothetical and non-parametric ridge functions and the multiple layer iteration method to enhance accuracy and solve the problems caused by the parameter selection and the subjective hypothesis. The NH-PPR model belongs to EDA and can be used to study high-dimensional data. The presented NH-PPR model can not only evaluate the thermal properties in this study, but also calculate mechanical properties of concrete or cement in future study.

## Author contribution statement

1 - Conceived and designed the experiments;

2 - Performed the experiments;

3 - Analyzed and interpreted the data;

4 - Contributed reagents, materials, analysis tools or data;

5 - Wrote the paper.

## Data availability statement

The data that support the findings of this study are available from the corresponding author upon reasonable request.

## Declaration of competing interest

The authors declare that they have no known competing financial interests or personal relationships that could have appeared to influence the work reported in this paper.
