# Supplementary material for: Non-hypothetical projection pursuit regression for the prediction of hydration heat of Portland-cement-based cementitious system
Source: Heliyon. 2023 Aug 28;9(9):e19471. doi: 10.1016/j.heliyon.2023.e19471 (PMC10480638; doi:10.1016/j.heliyon.2023.e19471)
Supplement: Multimedia component 1 [file mmc1.docx]

**Appendix Table A1**

| Mixtures | CF/m^2^•kg^-1^ | SCMs Content/% | | Mineral content /% | | | | Hydration heat/(J•g^-1^) | | | | | | |
| --- | --- | --- | --- | --- | --- | --- | --- | --- | --- | --- | --- | --- | --- | --- |
|  |  | FA | SL | C_3_S | C_2_S | C_3_A | C_4_AF | 1d | 2d | 3d | 4d | 5d | 6d | 7d |
| *Low heat Portland cement* 1 |  |  |  |  |  |  |  |  |  |  |  |  |  |  |
| L1_(100)_ | 320 | 0 | 0 | 34.2 | 40.4 | 2.8 | 16.6 | 167 | 199 | 218 | 231 | 242 | 251 | 257 |
| L1_(85)_F_(15)_ | 320 | 15 | 0 | 34.2 | 40.4 | 2.8 | 16.6 | 147 | 177 | 194 | 207 | 217 | 225 | 228 |
| L1_(75)_F_(25)_ | 320 | 25 | 0 | 34.2 | 40.4 | 2.8 | 16.6 | 135 | 165 | 181 | 194 | 202 | 208 | 211 |
| L1_(65)_F_(35)_ | 320 | 35 | 0 | 34.2 | 40.4 | 2.8 | 16.6 | 116 | 147 | 163 | 172 | 181 | 187 | 193 |
| L1_(55)_F_(45)_ | 320 | 45 | 0 | 34.2 | 40.4 | 2.8 | 16.6 | 101 | 131 | 147 | 159 | 168 | 173 | 176 |
| L1_(85)_S_(15)_ | 320 | 0 | 15 | 34.2 | 40.4 | 2.8 | 16.6 | 148 | 173 | 195 | 212 | 224 | 236 | 243 |
| L1_(75)_S_(25)_ | 320 | 0 | 25 | 34.2 | 40.4 | 2.8 | 16.6 | 134 | 163 | 182 | 199 | 212 | 222 | 226 |
| L1_(65)_S_(35)_ | 320 | 0 | 35 | 34.2 | 40.4 | 2.8 | 16.6 | 126 | 151 | 167 | 181 | 192 | 201 | 204 |
| L1_(55)_S_(45)_ | 320 | 0 | 45 | 34.2 | 40.4 | 2.8 | 16.6 | 110 | 141 | 160 | 170 | 175 | 179 | 182 |
| L1_(85)_F_(7.5)_S_(7.5)_ | 320 | 7.5 | 7.5 | 34.2 | 40.4 | 2.8 | 16.6 | 138 | 170 | 190 | 204 | 216 | 225 | 232 |
| L1_(75)_F_(12.5)_S_(12.5)_ | 320 | 12.5 | 12.5 | 34.2 | 40.4 | 2.8 | 16.6 | 126 | 158 | 175 | 188 | 198 | 207 | 213 |
| L1_(65)_F_(17.5)_S_(17.5)_ | 320 | 17.5 | 17.5 | 34.2 | 40.4 | 2.8 | 16.6 | 113 | 139 | 157 | 169 | 181 | 189 | 196 |
| L1_(55)_F_(22.5)_S_(22.5)_ | 320 | 22.5 | 22.5 | 34.2 | 40.4 | 2.8 | 16.6 | 104 | 131 | 146 | 159 | 169 | 175 | 180 |
| L1_(85)_F_(5)_S_(10)_ | 320 | 5 | 10 | 34.2 | 40.4 | 2.8 | 16.6 | 146 | 182 | 197 | 208 | 218 | 227 | 230 |
| L1_(75)_F_(8.3)_S_(16.7)_ | 320 | 8.3 | 16.7 | 34.2 | 40.4 | 2.8 | 16.6 | 131 | 156 | 173 | 185 | 198 | 208 | 217 |
| L1_(65)_F_(11.7)_S_(23.3)_ | 320 | 11.7 | 23.3 | 34.2 | 40.4 | 2.8 | 16.6 | 122 | 144 | 161 | 176 | 187 | 196 | 199 |
| L1_(55)_F_(15)_S_(30)_ | 320 | 15 | 30 | 34.2 | 40.4 | 2.8 | 16.6 | 104 | 128 | 139 | 149 | 160 | 170 | 178 |
| L1_(85)_F_(10)_S_(5)_ | 320 | 10 | 5 | 34.2 | 40.4 | 2.8 | 16.6 | 139 | 170 | 189 | 201 | 211 | 218 | 226 |
| L1_(75)_F_(16.7)_S_(8.3)_ | 320 | 16.7 | 8.3 | 34.2 | 40.4 | 2.8 | 16.6 | 125 | 157 | 173 | 184 | 191 | 198 | 202 |
| L1_(65)_F_(23.3)_S_(11.7)_ | 320 | 23.3 | 11.7 | 34.2 | 40.4 | 2.8 | 16.6 | 111 | 138 | 156 | 168 | 177 | 184 | 189 |
| L1_(55)_F_(30)_S_(15)_ | 320 | 30 | 15 | 34.2 | 40.4 | 2.8 | 16.6 | 97 | 125 | 140 | 150 | 155 | 160 | 164 |
| *Low heat Portland cement* 2 |  |  |  |  |  |  |  |  |  |  |  |  |  |  |
| L2_(100)_ | 316 | 0 | 0 | 30.1 | 44.8 | 3.2 | 14.7 | 147 | 175 | 196 | 212 | 226 | 238 | 249 |
| *Ordinary Portland cement* 1 |  |  |  |  |  |  |  |  |  |  |  |  |  |  |
| O1_(100)_ | 350 | 0 | 0 | 65.4 | 13.9 | 6.8 | 10 | 153 | 219 | 253 | 275 | 291 | 303 | 313 |
| O1_(70)_F_(30)_ | 350 | 30 | 0 | 65.4 | 13.9 | 6.8 | 10 | 135 | 190 | 219 | 245 | 263 | 276 | 283 |
| O1_(60)_F_(40)_ | 350 | 40 | 0 | 65.4 | 13.9 | 6.8 | 10 | 94 | 158 | 187 | 210 | 230 | 248 | 263 |
| O1_(50)_F_(50)_ | 350 | 50 | 0 | 65.4 | 13.9 | 6.8 | 10 | 74 | 133 | 162 | 184 | 204 | 222 | 238 |
| O1_(40)_F_(60)_ | 350 | 60 | 0 | 65.4 | 13.9 | 6.8 | 10 | 70 | 129 | 153 | 175 | 193 | 209 | 225 |
| O1_(70)_S_(30)_ | 350 | 0 | 30 | 65.4 | 13.9 | 6.8 | 10 | 136 | 190 | 224 | 246 | 266 | 282 | 292 |
| O1_(60)_S_(40)_ | 350 | 0 | 40 | 65.4 | 13.9 | 6.8 | 10 | 103 | 156 | 188 | 218 | 245 | 268 | 288 |
| O1_(50)_S_(50)_ | 350 | 0 | 50 | 65.4 | 13.9 | 6.8 | 10 | 100 | 153 | 185 | 213 | 238 | 258 | 275 |
| O1_(40)_S_(60)_ | 350 | 0 | 60 | 65.4 | 13.9 | 6.8 | 10 | 82 | 134 | 168 | 196 | 223 | 245 | 264 |
| O1_(30)_S_(70)_ | 350 | 0 | 70 | 65.4 | 13.9 | 6.8 | 10 | 92 | 133 | 165 | 189 | 210 | 227 | 241 |
| O1_(70)_F_(15)_S_(15)_ | 350 | 15 | 15 | 65.4 | 13.9 | 6.8 | 10 | 131 | 180 | 213 | 238 | 258 | 275 | 287 |
| O1_(60)_F_(20)_S_(20)_ | 350 | 20 | 20 | 65.4 | 13.9 | 6.8 | 10 | 123 | 177 | 207 | 232 | 249 | 264 | 276 |
| O1_(50)_F_(25)_S_(25)_ | 350 | 25 | 25 | 65.4 | 13.9 | 6.8 | 10 | 108 | 163 | 191 | 210 | 233 | 251 | 261 |
| *Ordinary Portland cement* 2 |  |  |  |  |  |  |  |  |  |  |  |  |  |  |
| O2_(100)_ | 358 | 0 | 0 | 64.2 | 15.8 | 6.1 | 10.8 | 162 | 230 | 261 | 281 | 295 | 306 | 314 |
| O2_(75)_F_(25)_ | 358 | 25 | 0 | 64.2 | 15.8 | 6.1 | 10.8 | 169 | 216 | 244 | 264 | 281 | 296 | 309 |
| O2_(70)_F_(30)_ | 358 | 35 | 0 | 64.2 | 15.8 | 6.1 | 10.8 | 138 | 191 | 219 | 243 | 259 | 272 | 283 |
| O2_(65)_F_(35)_ | 358 | 30 | 0 | 64.2 | 15.8 | 6.1 | 10.8 | 141 | 178 | 205 | 225 | 241 | 254 | 266 |
| O2_(60)_F_(40)_ | 358 | 40 | 0 | 64.2 | 15.8 | 6.1 | 10.8 | 110 | 164 | 193 | 215 | 235 | 250 | 260 |
| O2_(55)_F_(45)_ | 358 | 45 | 0 | 64.2 | 15.8 | 6.1 | 10.8 | 98 | 147 | 176 | 196 | 215 | 233 | 253 |
| O2_(50)_F_(50)_ | 358 | 50 | 0 | 64.2 | 15.8 | 6.1 | 10.8 | 98 | 139 | 164 | 184 | 204 | 222 | 237 |
| O2_(45)_F_(55)_ | 358 | 55 | 0 | 64.2 | 15.8 | 6.1 | 10.8 | 74 | 132 | 159 | 183 | 203 | 221 | 238 |
| O2_(40)_F_(60)_ | 358 | 60 | 0 | 64.2 | 15.8 | 6.1 | 10.8 | 70 | 128 | 153 | 175 | 193 | 209 | 225 |
| O2_(70)_S_(30)_ | 358 | 0 | 30 | 64.2 | 15.8 | 6.1 | 10.8 | 137 | 190 | 224 | 246 | 266 | 282 | 292 |
| O2_(60)_S_(40)_ | 358 | 0 | 40 | 64.2 | 15.8 | 6.1 | 10.8 | 103 | 157 | 187 | 216 | 243 | 267 | 286 |
| O2_(50)_S_(50)_ | 358 | 0 | 50 | 64.2 | 15.8 | 6.1 | 10.8 | 100 | 153 | 185 | 213 | 237 | 258 | 275 |
| O2_(40)_S_(60)_ | 358 | 0 | 60 | 64.2 | 15.8 | 6.1 | 10.8 | 83 | 136 | 168 | 195 | 220 | 239 | 256 |
| O2_(70)_F_(15)_S_(15)_ | 358 | 15 | 15 | 64.2 | 15.8 | 6.1 | 10.8 | 132 | 181 | 213 | 237 | 256 | 272 | 283 |
| O2_(60)_F_(20)_S_(20)_ | 358 | 20 | 20 | 64.2 | 15.8 | 6.1 | 10.8 | 123 | 177 | 207 | 232 | 249 | 264 | 276 |
| O2_(50)_F_(25)_S_(25)_ | 358 | 25 | 25 | 64.2 | 15.8 | 6.1 | 10.8 | 105 | 156 | 184 | 208 | 228 | 246 | 260 |
| *Moderate heat Portland cement* 1 |  |  |  |  |  |  |  |  |  |  |  |  |  |  |
| M1_(100)_ | 331 | 0 | 0 | 34.4 | 39 | 1.8 | 17.2 | 204 | 233 | 248 | 258 | 264 | 269 | 275 |
| *High sulfate_-_resistant Portland cement* 1 |  |  |  |  |  |  |  |  |  |  |  |  |  |  |
| H1_(100)_ | 364 | 0 | 0 | 37.8 | 35.6 | 0.1 | 18.51 | 211 | 243 | 257 | 268 | 278 | 285 | 295 |
| *High sulfate_-_resistant Portland cement* 2 |  |  |  |  |  |  |  |  |  |  |  |  |  |  |
| H2_(100)_ | 342 | 0 | 0 | 32.31 | 44.59 | 1.36 | 18.39 | 192 | 226 | 242 | 252 | 259 | 264 | 270 |
