# Supplementary material for: Non-hypothetical projection pursuit regression for the prediction of hydration heat of Portland-cement-based cementitious system
Source: Heliyon. 2023 Aug 28;9(9):e19471. doi: 10.1016/j.heliyon.2023.e19471 (PMC10480638; doi:10.1016/j.heliyon.2023.e19471)
Supplement: Multimedia component 2 [file mmc2.docx]

**Appendix Table A2**

| Mixtures | CF/m^2^•kg^-1^ | SCMs Content/% | | Mineral content /% | | | | Hydration heat/(J•g^-1^) | | | | | | |
| --- | --- | --- | --- | --- | --- | --- | --- | --- | --- | --- | --- | --- | --- | --- |
|  |  | FA | SL | C_3_S | C_2_S | C_3_A | C_4_AF | 1d | 2d | 3d | 4d | 5d | 6d | 7d |
| Published by Ren et al. [49] |  |  |  |  |  |  |  |  |  |  |  |  |  |  |
| Cement1 | 285 | 0 | 0 | 43.0 | 36.4 | 6.3 | 9.7 | - | - | 191 | - | - | - | 255 |
| Cement2 | 291 | 0 | 0 | 57.3 | 20.5 | 10.0 | 6.4 | - | - | 212 | - | - | - | 269 |
| Cement3 | 294 | 0 | 0 | 54.8 | 20.0 | 7.8 | 10.3 | - | - | 245 | - | - | - | 308 |
| Cement4 | 306 | 0 | 0 | 45.7 | 27.2 | 13.1 | 7.0 | - | - | 253 | - | - | - | 339 |
| Cement5 | 306 | 0 | 0 | 40.8 | 38.3 | 6.5 | 9.7 | - | - | 194 | - | - | - | 251 |
| Cement6 | 310 | 0 | 0 | 53.0 | 21.6 | 10.4 | 9.4 | - | - | 254 | - | - | - | 331 |
| Cement7 | 311 | 0 | 0 | 41.4 | 33.2 | 6.7 | 11.9 | - | - | 182 | - | - | - | 262 |
| Cement8 | 312 | 0 | 0 | 56.2 | 19.0 | 7.6 | 10.6 | - | - | 252 | - | - | - | 331 |
| Cement9 | 313 | 0 | 0 | 45.9 | 27.3 | 12.5 | 7.0 | - | - | 231 | - | - | - | 328 |
| Cement10 | 318 | 0 | 0 | 45.1 | 27.6 | 12.6 | 7.3 | - | - | 241 | - | - | - | 348 |
| Cement11 | 319 | 0 | 0 | 52.6 | 19.9 | 12.1 | 7.3 | - | - | 261 | - | - | - | 339 |
| Cement12 | 319 | 0 | 0 | 34.9 | 38.7 | 4.7 | 14.9 | - | - | 196 | - | - | - | 255 |
| Cement13 | 323 | 0 | 0 | 67.7 | 7.4 | 11.9 | 7.6 | - | - | 298 | - | - | - | 366 |
| Cement14 | 328 | 0 | 0 | 53.4 | 21.3 | 3.9 | 16.4 | - | - | 194 | - | - | - | 229 |
| Cement15 | 329 | 0 | 0 | 43.8 | 31.1 | 8.2 | 9.1 | - | - | 267 | - | - | - | 326 |
| Cement16 | 335 | 0 | 0 | 22.0 | 49.3 | 4.5 | 15.2 | - | - | 188 | - | - | - | 221 |
| Cement17 | 335 | 0 | 0 | 30.5 | 49.5 | 5.1 | 9.4 | - | - | 150 | - | - | - | 193 |
| Cement18 | 336 | 0 | 0 | 27.9 | 54.6 | 3.4 | 8.2 | - | - | 155 | - | - | - | 176 |
| Cement19 | 337 | 0 | 0 | 44.8 | 26.1 | 5.4 | 14.6 | - | - | 218 | - | - | - | 271 |
| Cement20 | 343 | 0 | 0 | 46.1 | 27.1 | 13.2 | 6.7 | - | - | 255 | - | - | - | 344 |
| Cement21 | 344 | 0 | 0 | 25.4 | 47.3 | 6.2 | 13.7 | - | - | 191 | - | - | - | 247 |
| Cement22 | 352 | 0 | 0 | 53.0 | 19.6 | 12.3 | 7.0 | - | - | 274 | - | - | - | 370 |
| Cement23 | 354 | 0 | 0 | 43.3 | 37.6 | 3.7 | 10.0 | - | - | 182 | - | - | - | 227 |
| Cement24 | 431 | 0 | 0 | 74.7 | 2.4 | 5.0 | 10.0 | - | - | 288 | - | - | - | 351 |
| Cement25 | 439 | 0 | 0 | 64.2 | 10.6 | 10.5 | 7.3 | - | - | 326 | - | - | - | 395 |
| Cement26 | 443 | 0 | 0 | 66.4 | 7.8 | 10.4 | 7.6 | - | - | 326 | - | - | - | 385 |
| Cement27 | 489 | 0 | 0 | 62.7 | 11.2 | 10.8 | 6.4 | - | - | 338 | - | - | - | 400 |
| Published by Li et al.[51] |  |  |  |  |  |  |  |  |  |  |  |  |  |  |
| Cement28 | 320 | 0 | 0 | 63.6 | 10.5 | 6.9 | 12.6 | - | - | 245 | - | - | - | 303 |
| Cement29 | 320 | 20 | 0 | 63.6 | 10.5 | 6.9 | 12.6 | - | - | 205 | - | - | - | 247 |
| Cement30 | 320 | 40 | 0 | 63.6 | 10.5 | 6.9 | 12.6 | - | - | 157 | - | - | - | 199 |
| moderate heat Portland cement, Published by Wu et al. [50] |  |  |  |  |  |  |  |  |  |  |  |  |  |  |
| M2_(100)_ | 330 | 0 | 0 | 55.5 | 21.6 | 3.9 | 16.7 | 162 | 205 | 228 | 241 | 252 | 258 | 261 |
| M2_(70)_F_(30)_ | 330 | 30 | 0 | 55.5 | 21.6 | 3.9 | 16.7 | 143 | 186 | 202 | 218 | 225 | 232 | 235 |
| M2_(60)_F_(40)_ | 330 | 40 | 0 | 55.5 | 21.6 | 3.9 | 16.7 | 115 | 168 | 190 | 210 | 218 | 227 | 231 |
| M2_(50)_F_(50)_ | 330 | 50 | 0 | 55.5 | 21.6 | 3.9 | 16.7 | 112 | 160 | 181 | 198 | 207 | 214 | 220 |
| M2_(40)_F_(60)_ | 330 | 60 | 0 | 55.5 | 21.6 | 3.9 | 16.7 | 82 | 125 | 148 | 161 | 173 | 184 | 189 |
| M2_(30)_F_(70)_ | 330 | 70 | 0 | 55.5 | 21.6 | 3.9 | 16.7 | 67 | 110 | 134 | 150 | 161 | 166 | 171 |
| M2_(70)_S_(30)_ | 330 | 0 | 30 | 55.5 | 21.6 | 3.9 | 16.7 | 25 | 168 | 198 | 213 | 224 | 235 | 244 |
| M2_(60)_S_(40)_ | 330 | 0 | 40 | 55.5 | 21.6 | 3.9 | 16.7 | 105 | 147 | 171 | 187 | 201 | 212 | 221 |
| M2_(50)_S_(50)_ | 330 | 0 | 50 | 55.5 | 21.6 | 3.9 | 16.7 | 102 | 140 | 158 | 169 | 178 | 187 | 195 |
| M2_(40)_S_(60)_ | 330 | 0 | 60 | 55.5 | 21.6 | 3.9 | 16.7 | 88 | 118 | 134 | 142 | 148 | 153 | 160 |
| M2_(30)_S_(70)_ | 330 | 0 | 70 | 55.5 | 21.6 | 3.9 | 16.7 | 72 | 99 | 109 | 115 | 120 | 124 | 128 |
| M2_(70)_F_(12)_S_(18)_ | 330 | 12 | 18 | 55.5 | 21.6 | 3.9 | 16.7 | 136 | 193 | 209 | 230 | 240 | 248 | 258 |
| M2_(60)_F_(16)_S_(24)_ | 330 | 16 | 24 | 55.5 | 21.6 | 3.9 | 16.7 | 107 | 150 | 175 | 190 | 205 | 215 | 226 |
| M2_(50)_F_(20)_S_(30)_ | 330 | 20 | 30 | 55.5 | 21.6 | 3.9 | 16.7 | 101 | 147 | 173 | 195 | 210 | 224 | 235 |
| M2_(40)_F_(24)_S_(36)_ | 330 | 24 | 36 | 55.5 | 21.6 | 3.9 | 16.7 | 85 | 133 | 158 | 181 | 195 | 209 | 219 |
| M2_(30)_F_(28)_S_(42)_ | 330 | 28 | 42 | 55.5 | 21.6 | 3.9 | 16.7 | 72 | 119 | 148 | 160 | 174 | 186 | 194 |
