# Supplementary material for: Non-hypothetical projection pursuit regression for the prediction of hydration heat of Portland-cement-based cementitious system
Source: Heliyon. 2023 Aug 28;9(9):e19471. doi: 10.1016/j.heliyon.2023.e19471 (PMC10480638; doi:10.1016/j.heliyon.2023.e19471)
Supplement: Multimedia component 3 [file mmc3.docx]

**Appendix Table A3**

| Mixtures | CF/m^2^•kg^-1^ | SCMs Content/% | | Mineral content /% | | | | Hydration heat/(J•g^-1^) | | | | | | |
| --- | --- | --- | --- | --- | --- | --- | --- | --- | --- | --- | --- | --- | --- | --- |
|  |  | FA | SL | C_3_S | C_2_S | C_3_A | C_4_AF | 1d | 2d | 3d | 4d | 5d | 6d | 7d |
| Published by Li et al. [52] |  |  |  |  |  |  |  |  |  |  |  |  |  |  |
| M3_(60)_F_(10)_S_(30)_ | 370 | 10 | 30 | 48.0 | 24.8 | 8.4 | 10.2 | 193 | 224 | 239 | 245 | 249 | 252 | 254 |
| M3_(60)_F_(20)_S_(30)_ | 370 | 20 | 30 | 48.0 | 24.8 | 8.4 | 10.2 | 119 | 190 | 208 | 220 | 230 | 238 | 244 |
| M3_(60)_F_(20)_S_(40)_ | 370 | 20 | 40 | 48.0 | 24.8 | 8.4 | 10.2 | 91 | 168 | 191 | 190 | 206 | 208 | 210 |
