# Supplementary material for: Non-hypothetical projection pursuit regression for the prediction of hydration heat of Portland-cement-based cementitious system
Source: Heliyon. 2023 Aug 28;9(9):e19471. doi: 10.1016/j.heliyon.2023.e19471 (PMC10480638; doi:10.1016/j.heliyon.2023.e19471)
Supplement: Multimedia component 4 [file mmc4.docx]

**Appendix Table A4**

| ***f*_1_** | | ***f*_2_** | | ***f*_3_** | |
| --- | --- | --- | --- | --- | --- |
| ***X*_1_** | ***Y*_1_** | ***X*_2_** | ***Y*_2_** | ***X*_3_** | ***Y*_3_** |
| \| 0.400855 \| \| --- \| \| 0.728334 \| \| 0.733967 \| \| 0.892074 \| \| 1.245438 \| \| 1.383293 \| \| 1.650909 \| \| 1.664578 \| \| 1.736657 \| \| 1.920012 \| \| 2.046637 \| \| 2.090021 \| \| 2.173262 \| \| 2.262203 \| \| 2.423133 \| \| 2.428696 \| \| 2.495492 \| \| 2.509161 \| \| 2.58124 \| \| 2.74498 \| \| 2.764595 \| \| 2.78364 \| \| 2.842273 \| \| 2.89122 \| \| 2.928301 \| \| 2.934604 \| \| 3.017845 \| \| 3.106786 \| \| 3.109506 \| \| 3.118387 \| \| 3.261413 \| \| 3.267716 \| \| 3.272115 \| \| 3.273279 \| \| 3.292895 \| \| 3.307607 \| \| 3.353744 \| \| 3.4741 \| \| 3.589563 \| \| 3.609178 \| \| 3.686856 \| \| 3.750248 \| \| 3.779187 \| \| 3.862429 \| \| 3.894341 \| \| 3.917042 \| \| 3.951369 \| \| 3.954089 \| \| 3.96297 \| \| 4.045783 \| \| 4.105996 \| \| 4.106667 \| \| 4.112299 \| \| 4.116698 \| \| 4.137478 \| \| 4.184658 \| \| 4.192024 \| \| 4.198327 \| \| 4.270406 \| \| 4.301184 \| \| 4.318683 \| \| 4.35301 \| \| 4.428303 \| \| 4.434146 \| \| 4.453761 \| \| 4.472806 \| \| 4.519503 \| \| 4.525136 \| \| 4.531439 \| \| 4.580386 \| \| 4.582032 \| \| 4.623771 \| \| 4.760954 \| \| 4.795952 \| \| 4.798672 \| \| 4.807553 \| \| 4.890366 \| \| 4.950579 \| \| 4.95125 \| \| 4.956882 \| \| 4.961282 \| \| 4.962445 \| \| 4.982061 \| \| 4.996773 \| \| 5.029241 \| \| 5.036607 \| \| 5.04291 \| \| 5.049103 \| \| 5.081637 \| \| 5.145767 \| \| 5.163266 \| \| 5.197593 \| \| 5.278729 \| \| 5.317389 \| \| 5.369719 \| \| 5.376022 \| \| 5.398414 \| \| 5.424969 \| \| 5.426615 \| \| 5.439414 \| \| 5.468354 \| \| 5.473707 \| \| 5.605537 \| \| 5.627435 \| \| 5.640535 \| \| 5.643255 \| \| 5.645833 \| \| 5.73495 \| \| 5.751005 \| \| 5.772491 \| \| 5.788859 \| \| 5.795162 \| \| 5.795833 \| \| 5.799561 \| \| 5.801466 \| \| 5.805865 \| \| 5.812218 \| \| 5.826644 \| \| 5.841356 \| \| 5.858554 \| \| 5.873824 \| \| 5.88119 \| \| 5.887493 \| \| 5.92622 \| \| 5.959572 \| \| 5.99035 \| \| 6.042176 \| \| 6.064479 \| \| 6.070378 \| \| 6.082472 \| \| 6.087608 \| \| 6.11747 \| \| 6.123312 \| \| 6.161972 \| \| 6.181834 \| \| 6.208669 \| \| 6.220605 \| \| 6.2362 \| \| 6.242997 \| \| 6.269552 \| \| 6.271198 \| \| 6.277694 \| \| 6.283998 \| \| 6.31829 \| \| 6.396178 \| \| 6.443817 \| \| 6.45012 \| \| 6.471952 \| \| 6.472018 \| \| 6.487838 \| \| 6.490416 \| \| 6.496719 \| \| 6.51911 \| \| 6.617075 \| \| 6.633442 \| \| 6.639745 \| \| 6.644144 \| \| 6.650448 \| \| 6.651611 \| \| 6.656801 \| \| 6.671227 \| \| 6.672839 \| \| 6.685939 \| \| 6.703137 \| \| 6.713498 \| \| 6.718407 \| \| 6.725773 \| \| 6.732077 \| \| 6.738269 \| \| 6.770803 \| \| 6.834933 \| \| 6.852432 \| \| 6.889513 \| \| 6.914961 \| \| 6.927055 \| \| 6.932191 \| \| 6.944093 \| \| 6.98751 \| \| 7.006555 \| \| 7.053252 \| \| 7.058885 \| \| 7.063284 \| \| 7.065188 \| \| 7.069588 \| \| 7.080783 \| \| 7.114136 \| \| 7.115781 \| \| 7.122277 \| \| 7.162873 \| \| 7.237035 \| \| 7.272673 \| \| 7.306433 \| \| 7.316535 \| \| 7.316602 \| \| 7.332422 \| \| 7.341302 \| \| 7.363694 \| \| 7.390763 \| \| 7.414068 \| \| 7.424116 \| \| 7.461658 \| \| 7.478025 \| \| 7.484328 \| \| 7.488727 \| \| 7.495031 \| \| 7.496195 \| \| 7.501384 \| \| 7.51581 \| \| 7.517422 \| \| 7.530522 \| \| 7.547721 \| \| 7.570356 \| \| 7.582852 \| \| 7.595805 \| \| 7.679516 \| \| 7.697015 \| \| 7.701562 \| \| 7.718242 \| \| 7.731342 \| \| 7.734096 \| \| 7.742894 \| \| 7.754139 \| \| 7.759544 \| \| 7.771638 \| \| 7.776775 \| \| 7.788676 \| \| 7.806636 \| \| 7.851138 \| \| 7.871 \| \| 7.897835 \| \| 7.903468 \| \| 7.907867 \| \| 7.914171 \| \| 7.925366 \| \| 7.932163 \| \| 7.96686 \| \| 7.969448 \| \| 7.973164 \| \| 7.995799 \| \| 8.007456 \| \| 8.132983 \| \| 8.139287 \| \| 8.161118 \| \| 8.161185 \| \| 8.177005 \| \| 8.179582 \| \| 8.185885 \| \| 8.208277 \| \| 8.214003 \| \| 8.235346 \| \| 8.268699 \| \| 8.322608 \| \| 8.328911 \| \| 8.33331 \| \| 8.339614 \| \| 8.345967 \| \| 8.360393 \| \| 8.362005 \| \| 8.386 \| \| 8.390269 \| \| 8.392304 \| \| 8.409097 \| \| 8.414939 \| \| 8.427435 \| \| 8.440388 \| \| 8.524099 \| \| 8.541598 \| \| 8.546145 \| \| 8.562826 \| \| 8.575925 \| \| 8.578679 \| \| 8.598722 \| \| 8.604127 \| \| 8.616221 \| \| 8.621358 \| \| 8.633259 \| \| 8.651219 \| \| 8.715583 \| \| 8.748051 \| \| 8.75245 \| \| 8.758754 \| \| 8.769949 \| \| 8.776746 \| \| 8.811443 \| \| 8.817747 \| \| 8.852039 \| \| 8.926201 \| \| 8.958766 \| \| 8.977566 \| \| 8.98387 \| \| 9.005701 \| \| 9.005768 \| \| 9.024165 \| \| 9.05286 \| \| 9.079929 \| \| 9.129337 \| \| 9.150824 \| \| 9.167191 \| \| 9.177894 \| \| 9.19055 \| \| 9.206588 \| \| 9.230583 \| \| 9.236887 \| \| 9.25368 \| \| 9.259522 \| \| 9.272018 \| \| 9.304552 \| \| 9.368682 \| \| 9.390728 \| \| 9.407409 \| \| 9.420508 \| \| 9.423262 \| \| 9.443305 \| \| 9.449608 \| \| 9.460804 \| \| 9.465941 \| \| 9.477842 \| \| 9.495802 \| \| 9.560166 \| \| 9.580028 \| \| 9.587002 \| \| 9.597033 \| \| 9.603337 \| \| 9.614532 \| \| 9.621329 \| \| 9.656027 \| \| 9.696622 \| \| 9.770784 \| \| 9.784677 \| \| 9.822149 \| \| 9.850351 \| \| 9.868748 \| \| 9.897443 \| \| 9.924513 \| \| 9.995407 \| \| 10.02248 \| \| 10.03513 \| \| 10.05117 \| \| 10.07517 \| \| 10.09183 \| \| 10.09546 \| \| 10.09826 \| \| 10.1166 \| \| 10.12955 \| \| 10.23531 \| \| 10.25199 \| \| 10.28789 \| \| 10.30539 \| \| 10.31052 \| \| 10.40475 \| \| 10.42461 \| \| 10.44162 \| \| 10.45912 \| \| 10.49689 \| \| 10.50061 \| \| 10.50377 \| \| 10.54121 \| \| 10.5965 \| \| 10.61537 \| \| 10.66673 \| \| 10.69487 \| \| 10.69493 \| \| 10.71333 \| \| 10.74203 \| \| 10.7691 \| \| 10.7924 \| \| 10.85445 \| \| 10.85636 \| \| 10.89575 \| \| 10.91975 \| \| 10.94285 \| \| 10.97414 \| \| 11.07989 \| \| 11.09657 \| \| 11.12123 \| \| 11.13247 \| \| 11.14997 \| \| 11.15511 \| \| 11.15527 \| \| 11.17506 \| \| 11.23108 \| \| 11.24933 \| \| 11.26919 \| \| 11.2862 \| \| 11.3037 \| \| 11.32364 \| \| 11.34519 \| \| 11.34778 \| \| 11.41043 \| \| 11.45995 \| \| 11.51132 \| \| 11.53945 \| \| 11.58661 \| \| 11.5945 \| \| 11.61368 \| \| 11.69904 \| \| 11.74034 \| \| 11.76433 \| \| 11.7686 \| \| 11.92448 \| \| 11.94116 \| \| 11.97705 \| \| 12.11378 \| \| 12.13078 \| \| 12.30453 \| \| 12.3371 \| \| 12.352 \| \| 12.45826 \| \| 12.54362 \| \| 12.76906 \| \| 12.78574 \| \| 13.02434 \| \| 13.23447 \| \| 13.3882 \| \| 13.47379 \| \| 13.51766 \| \| 13.80294 \| \| 13.87523 \| \| 13.8821 \| \| 14.07906 \| \| 14.23279 \| \| 14.5336 \| \| 14.55339 \| \| 14.63533 \| \| 14.64753 \| \| 14.70197 \| \| 14.78876 \| \| 14.92364 \| \| 14.97283 \| \| 15.07737 \| \| 15.73033 \| \| 15.76822 \| \| 15.79434 \| \| 15.92195 \| \| 16.40267 \| \| 16.89599 \| \| 16.96686 \| \| 17.56606 \| \| 17.71259 \| \| 18.42499 \| \| 19.72696 \| \| 20.3452 \| \| 20.94439 \| \| 21.80332 \| | \| -125.644 \| \| --- \| \| -120.553 \| \| -120.466 \| \| -118.007 \| \| -112.514 \| \| -110.37 \| \| -106.21 \| \| -105.997 \| \| -104.877 \| \| -102.026 \| \| -100.057 \| \| -99.383 \| \| -98.0888 \| \| -96.7061 \| \| -94.2041 \| \| -94.1176 \| \| -93.0791 \| \| -92.8666 \| \| -91.746 \| \| -89.2003 \| \| -88.8954 \| \| -88.5993 \| \| -87.6877 \| \| -86.9267 \| \| -86.3503 \| \| -86.2523 \| \| -84.9581 \| \| -83.5753 \| \| -83.5331 \| \| -83.395 \| \| -81.1714 \| \| -81.0734 \| \| -81.005 \| \| -80.9869 \| \| -80.6819 \| \| -80.4532 \| \| -79.7359 \| \| -77.8647 \| \| -76.0696 \| \| -75.7647 \| \| -74.557 \| \| -73.5715 \| \| -73.1215 \| \| -71.8274 \| \| -71.3313 \| \| -70.9783 \| \| -70.4446 \| \| -70.4023 \| \| -70.2643 \| \| -68.9768 \| \| -68.0407 \| \| -68.0302 \| \| -67.9427 \| \| -67.8743 \| \| -67.5512 \| \| -66.8177 \| \| -66.7032 \| \| -66.6052 \| \| -65.4846 \| \| -65.0061 \| \| -64.734 \| \| -64.2003 \| \| -63.0298 \| \| -62.9389 \| \| -62.634 \| \| -62.3379 \| \| -61.6119 \| \| -61.5243 \| \| -61.4187 \| \| -60.6477 \| \| -60.6175 \| \| -59.9581 \| \| -57.7875 \| \| -57.2275 \| \| -57.1782 \| \| -57.0347 \| \| -55.7106 \| \| -54.7408 \| \| -54.7243 \| \| -54.6284 \| \| -54.5528 \| \| -54.5303 \| \| -54.209 \| \| -53.9682 \| \| -53.4374 \| \| -53.3174 \| \| -53.2156 \| \| -53.1145 \| \| -52.5795 \| \| -51.5207 \| \| -51.2319 \| \| -50.6641 \| \| -49.3161 \| \| -48.6709 \| \| -47.7947 \| \| -47.6894 \| \| -47.3129 \| \| -46.8643 \| \| -46.8373 \| \| -46.6229 \| \| -46.1344 \| \| -46.0485 \| \| -43.7991 \| \| -43.4231 \| \| -43.1976 \| \| -43.1481 \| \| -43.1004 \| \| -41.5489 \| \| -41.2601 \| \| -40.8756 \| \| -40.5809 \| \| -40.4546 \| \| -40.4277 \| \| -40.3495 \| \| -40.3056 \| \| -40.2175 \| \| -40.096 \| \| -39.8293 \| \| -39.5557 \| \| -39.237 \| \| -38.9529 \| \| -38.8128 \| \| -38.6934 \| \| -37.9851 \| \| -37.3716 \| \| -36.8068 \| \| -35.8535 \| \| -35.4403 \| \| -35.3282 \| \| -35.1032 \| \| -35.0055 \| \| -34.4497 \| \| -34.3398 \| \| -33.6169 \| \| -33.2428 \| \| -32.7388 \| \| -32.515 \| \| -32.2201 \| \| -32.0921 \| \| -31.5919 \| \| -31.5596 \| \| -31.4363 \| \| -31.3194 \| \| -30.6688 \| \| -29.1856 \| \| -28.2751 \| \| -28.1549 \| \| -27.7395 \| \| -27.7439 \| \| -27.45 \| \| -27.409 \| \| -27.2975 \| \| -26.8753 \| \| -24.9948 \| \| -24.6873 \| \| -24.5744 \| \| -24.4982 \| \| -24.3857 \| \| -24.3723 \| \| -24.2828 \| \| -24.013 \| \| -23.9921 \| \| -23.7444 \| \| -23.413 \| \| -23.2137 \| \| -23.1222 \| \| -22.9855 \| \| -22.8691 \| \| -22.7565 \| \| -22.1282 \| \| -20.8808 \| \| -20.5446 \| \| -19.8256 \| \| -19.335 \| \| -19.1065 \| \| -19.0144 \| \| -18.79 \| \| -17.948 \| \| -17.5816 \| \| -16.6763 \| \| -16.5744 \| \| -16.4975 \| \| -16.4704 \| \| -16.3932 \| \| -16.1799 \| \| -15.5358 \| \| -15.5108 \| \| -15.3903 \| \| -14.605 \| \| -13.1625 \| \| -12.4726 \| \| -11.8213 \| \| -11.6323 \| \| -11.6432 \| \| -11.3446 \| \| -11.1808 \| \| -10.7521 \| \| -10.2307 \| \| -9.78316 \| \| -9.59251 \| \| -8.86499 \| \| -8.55006 \| \| -8.43255 \| \| -8.35312 \| \| -8.23477 \| \| -8.21505 \| \| -8.11721 \| \| -7.83942 \| \| -7.80903 \| \| -7.55556 \| \| -7.21887 \| \| -6.77537 \| \| -6.52804 \| \| -6.26999 \| \| -4.64478 \| \| -4.30044 \| \| -4.20822 \| \| -3.88117 \| \| -3.62303 \| \| -3.56461 \| \| -3.38865 \| \| -3.16176 \| \| -3.04869 \| \| -2.80709 \| \| -2.70046 \| \| -2.46348 \| \| -2.10856 \| \| -1.24208 \| \| -0.85292 \| \| -0.33167 \| \| -0.21649 \| \| -0.12682 \| \| -0.00432 \| \| 0.215357 \| \| 0.351382 \| \| 1.017973 \| \| 1.076005 \| \| 1.156562 \| \| 1.594402 \| \| 1.824949 \| \| 4.192076 \| \| 4.314973 \| \| 4.725865 \| \| 4.726723 \| \| 5.021657 \| \| 5.072179 \| \| 5.187327 \| \| 5.602423 \| \| 5.70864 \| \| 6.1083 \| \| 6.729535 \| \| 7.730754 \| \| 7.844133 \| \| 7.918943 \| \| 8.028764 \| \| 8.138684 \| \| 8.398446 \| \| 8.421248 \| \| 8.861545 \| \| 8.932143 \| \| 8.965921 \| \| 9.274976 \| \| 9.380508 \| \| 9.608669 \| \| 9.84625 \| \| 11.39055 \| \| 11.71125 \| \| 11.7918 \| \| 12.09415 \| \| 12.32965 \| \| 12.37593 \| \| 12.74018 \| \| 12.83318 \| \| 13.04956 \| \| 13.13847 \| \| 13.35309 \| \| 13.67948 \| \| 14.85649 \| \| 15.44485 \| \| 15.51812 \| \| 15.62406 \| \| 15.82202 \| \| 15.93827 \| \| 16.56346 \| \| 16.67409 \| \| 17.28996 \| \| 18.62486 \| \| 19.20691 \| \| 19.54667 \| \| 19.66046 \| \| 20.05006 \| \| 20.04645 \| \| 20.3727 \| \| 20.88209 \| \| 21.36503 \| \| 22.244 \| \| 22.62705 \| \| 22.91761 \| \| 23.10463 \| \| 23.32586 \| \| 23.60687 \| \| 24.03093 \| \| 24.14025 \| \| 24.43548 \| \| 24.53802 \| \| 24.75863 \| \| 25.32973 \| \| 26.45143 \| \| 26.83726 \| \| 27.12909 \| \| 27.3589 \| \| 27.40996 \| \| 27.76123 \| \| 27.87339 \| \| 28.0721 \| \| 28.16627 \| \| 28.37872 \| \| 28.69586 \| \| 29.8087 \| \| 30.15297 \| \| 30.27871 \| \| 30.45647 \| \| 30.56998 \| \| 30.76843 \| \| 30.89211 \| \| 31.49025 \| \| 32.18887 \| \| 33.45503 \| \| 33.70093 \| \| 34.34329 \| \| 34.82817 \| \| 35.14743 \| \| 35.63832 \| \| 36.10003 \| \| 37.29345 \| \| 37.75089 \| \| 37.96754 \| \| 38.24072 \| \| 38.64762 \| \| 38.93122 \| \| 38.99806 \| \| 39.05242 \| \| 39.36422 \| \| 39.58731 \| \| 41.3354 \| \| 41.61267 \| \| 42.20336 \| \| 42.49213 \| \| 42.57864 \| \| 44.11995 \| \| 44.44519 \| \| 44.7247 \| \| 45.01235 \| \| 45.62836 \| \| 45.68942 \| \| 45.74165 \| \| 46.34642 \| \| 47.23998 \| \| 47.54423 \| \| 48.37429 \| \| 48.82928 \| \| 48.83059 \| \| 49.1266 \| \| 49.58918 \| \| 50.02705 \| \| 50.40462 \| \| 51.40647 \| \| 51.44656 \| \| 52.08531 \| \| 52.47802 \| \| 52.85575 \| \| 53.36475 \| \| 55.06272 \| \| 55.33693 \| \| 55.73827 \| \| 55.92636 \| \| 56.21436 \| \| 56.29616 \| \| 56.29875 \| \| 56.61393 \| \| 57.50608 \| \| 57.79668 \| \| 58.11297 \| \| 58.38379 \| \| 58.66246 \| \| 58.98002 \| \| 59.32324 \| \| 59.36444 \| \| 60.3621 \| \| 61.15074 \| \| 61.96872 \| \| 62.41677 \| \| 63.16775 \| \| 63.29335 \| \| 63.59883 \| \| 64.95814 \| \| 65.61585 \| \| 65.99797 \| \| 66.06595 \| \| 68.54824 \| \| 68.81388 \| \| 69.38552 \| \| 71.56281 \| \| 71.83362 \| \| 74.60057 \| \| 75.11917 \| \| 75.35642 \| \| 77.04867 \| \| 78.40797 \| \| 81.99808 \| \| 82.26372 \| \| 86.06336 \| \| 89.40971 \| \| 91.85781 \| \| 93.22081 \| \| 93.91937 \| \| 98.46248 \| \| 99.61357 \| \| 99.72301 \| \| 102.8595 \| \| 105.3076 \| \| 110.0981 \| \| 110.4133 \| \| 111.718 \| \| 111.9123 \| \| 112.7794 \| \| 114.1614 \| \| 116.3094 \| \| 117.0927 \| \| 118.7575 \| \| 129.1558 \| \| 129.7592 \| \| 130.1751 \| \| 132.2073 \| \| 139.8627 \| \| 147.7187 \| \| 148.8474 \| \| 158.3895 \| \| 160.7229 \| \| 172.0677 \| \| 192.8014 \| \| 202.6467 \| \| 212.1888 \| \| 225.867 \| | \| -12.0093 \| \| --- \| \| -11.7347 \| \| -11.7166 \| \| -11.7067 \| \| -11.6649 \| \| -11.5951 \| \| -11.56 \| \| -11.5329 \| \| -11.5286 \| \| -11.5253 \| \| -11.525 \| \| -11.4984 \| \| -11.4726 \| \| -11.467 \| \| -11.461 \| \| -11.4494 \| \| -11.4379 \| \| -11.4205 \| \| -11.4087 \| \| -11.4005 \| \| -11.3914 \| \| -11.3852 \| \| -11.3505 \| \| -11.3038 \| \| -11.2571 \| \| -11.2092 \| \| -11.0845 \| \| -10.8 \| \| -10.7405 \| \| -10.7125 \| \| -10.6707 \| \| -10.5657 \| \| -10.5616 \| \| -10.5387 \| \| -10.5311 \| \| -10.5308 \| \| -10.5042 \| \| -10.4917 \| \| -10.4728 \| \| -10.4668 \| \| -10.4552 \| \| -10.4437 \| \| -10.4263 \| \| -10.4219 \| \| -10.4145 \| \| -10.4063 \| \| -10.3992 \| \| -10.391 \| \| -10.3703 \| \| -10.3217 \| \| -10.3096 \| \| -10.2914 \| \| -10.2823 \| \| -10.2629 \| \| -10.2149 \| \| -10.1066 \| \| -10.0728 \| \| -10.0726 \| \| -10.0725 \| \| -10.0724 \| \| -10.0723 \| \| -10.0722 \| \| -10.0713 \| \| -9.93274 \| \| -9.83936 \| \| -9.74631 \| \| -9.71825 \| \| -9.67649 \| \| -9.66253 \| \| -9.62762 \| \| -9.59271 \| \| -9.57154 \| \| -9.56736 \| \| -9.5445 \| \| -9.53685 \| \| -9.52289 \| \| -9.50996 \| \| -9.49754 \| \| -9.48798 \| \| -9.48415 \| \| -9.47859 \| \| -9.47259 \| \| -9.45307 \| \| -9.44946 \| \| -9.43212 \| \| -9.42772 \| \| -9.42033 \| \| -9.41209 \| \| -9.39677 \| \| -9.39179 \| \| -9.36208 \| \| -9.3579 \| \| -9.35466 \| \| -9.34934 \| \| -9.32196 \| \| -9.31538 \| \| -9.30778 \| \| -9.30143 \| \| -9.28986 \| \| -9.2783 \| \| -9.24361 \| \| -9.22844 \| \| -9.18232 \| \| -9.14825 \| \| -9.1009 \| \| -9.08193 \| \| -9.07861 \| \| -9.07835 \| \| -9.07826 \| \| -9.07818 \| \| -9.07809 \| \| -9.078 \| \| -9.07746 \| \| -9.05684 \| \| -9.03298 \| \| -9.01912 \| \| -9.01014 \| \| -9.00755 \| \| -8.98548 \| \| -8.97286 \| \| -8.96345 \| \| -8.96126 \| \| -8.93854 \| \| -8.90912 \| \| -8.89184 \| \| -8.8748 \| \| -8.84515 \| \| -8.83278 \| \| -8.82162 \| \| -8.81717 \| \| -8.79846 \| \| -8.78609 \| \| -8.7521 \| \| -8.7394 \| \| -8.72405 \| \| -8.68228 \| \| -8.66832 \| \| -8.64601 \| \| -8.63341 \| \| -8.61434 \| \| -8.57315 \| \| -8.56359 \| \| -8.55029 \| \| -8.54596 \| \| -8.54264 \| \| -8.54243 \| \| -8.52868 \| \| -8.51575 \| \| -8.50333 \| \| -8.49377 \| \| -8.48438 \| \| -8.47838 \| \| -8.46682 \| \| -8.45886 \| \| -8.45526 \| \| -8.43791 \| \| -8.43351 \| \| -8.42613 \| \| -8.42395 \| \| -8.41788 \| \| -8.40256 \| \| -8.39758 \| \| -8.36787 \| \| -8.36369 \| \| -8.32776 \| \| -8.30722 \| \| -8.29566 \| \| -8.29387 \| \| -8.28409 \| \| -8.27449 \| \| -8.25794 \| \| -8.2494 \| \| -8.22779 \| \| -8.22654 \| \| -8.18812 \| \| -8.08415 \| \| -8.08406 \| \| -8.08388 \| \| -8.0838 \| \| -8.06263 \| \| -8.03648 \| \| -8.03252 \| \| -8.02491 \| \| -8.01594 \| \| -8.01335 \| \| -7.97866 \| \| -7.96924 \| \| -7.9523 \| \| -7.94433 \| \| -7.89764 \| \| -7.85094 \| \| -7.83858 \| \| -7.80425 \| \| -7.79188 \| \| -7.7579 \| \| -7.75756 \| \| -7.74519 \| \| -7.73976 \| \| -7.72984 \| \| -7.71997 \| \| -7.68808 \| \| -7.67412 \| \| -7.65181 \| \| -7.57895 \| \| -7.56939 \| \| -7.55608 \| \| -7.55175 \| \| -7.54843 \| \| -7.54822 \| \| -7.53448 \| \| -7.52155 \| \| -7.50912 \| \| -7.49957 \| \| -7.49574 \| \| -7.49018 \| \| -7.47261 \| \| -7.46466 \| \| -7.4437 \| \| -7.4393 \| \| -7.42975 \| \| -7.42368 \| \| -7.4166 \| \| -7.4146 \| \| -7.40836 \| \| -7.40337 \| \| -7.37366 \| \| -7.36948 \| \| -7.33355 \| \| -7.32697 \| \| -7.30145 \| \| -7.29966 \| \| -7.28989 \| \| -7.26373 \| \| -7.25519 \| \| -7.23233 \| \| -7.19391 \| \| -7.11512 \| \| -7.10765 \| \| -7.0902 \| \| -7.08994 \| \| -7.08985 \| \| -7.08976 \| \| -7.08968 \| \| -7.08959 \| \| -7.06842 \| \| -7.03071 \| \| -7.02173 \| \| -7.01914 \| \| -6.98445 \| \| -6.97504 \| \| -6.95012 \| \| -6.90343 \| \| -6.84437 \| \| -6.82317 \| \| -6.81005 \| \| -6.79768 \| \| -6.76369 \| \| -6.76335 \| \| -6.75099 \| \| -6.73564 \| \| -6.69387 \| \| -6.6576 \| \| -6.645 \| \| -6.58474 \| \| -6.57518 \| \| -6.56188 \| \| -6.55755 \| \| -6.55423 \| \| -6.55402 \| \| -6.54027 \| \| -6.52734 \| \| -6.51492 \| \| -6.50536 \| \| -6.50153 \| \| -6.49597 \| \| -6.48997 \| \| -6.47841 \| \| -6.47045 \| \| -6.4495 \| \| -6.4451 \| \| -6.43554 \| \| -6.42947 \| \| -6.42239 \| \| -6.40917 \| \| -6.33935 \| \| -6.33277 \| \| -6.31881 \| \| -6.31453 \| \| -6.30724 \| \| -6.30546 \| \| -6.29568 \| \| -6.28607 \| \| -6.26953 \| \| -6.26099 \| \| -6.1997 \| \| -6.12973 \| \| -6.12091 \| \| -6.09599 \| \| -6.09573 \| \| -6.09565 \| \| -6.09556 \| \| -6.09547 \| \| -6.09444 \| \| -6.07422 \| \| -6.0365 \| \| -6.02752 \| \| -6.02493 \| \| -5.99825 \| \| -5.99024 \| \| -5.95592 \| \| -5.86253 \| \| -5.85016 \| \| -5.81584 \| \| -5.80347 \| \| -5.79922 \| \| -5.76948 \| \| -5.76915 \| \| -5.75678 \| \| -5.74143 \| \| -5.71009 \| \| -5.69966 \| \| -5.6857 \| \| -5.66339 \| \| -5.65079 \| \| -5.62984 \| \| -5.59471 \| \| -5.58097 \| \| -5.56334 \| \| -5.56002 \| \| -5.55981 \| \| -5.53314 \| \| -5.52071 \| \| -5.51115 \| \| -5.50733 \| \| -5.50176 \| \| -5.49576 \| \| -5.47624 \| \| -5.45529 \| \| -5.45089 \| \| -5.44133 \| \| -5.43526 \| \| -5.42819 \| \| -5.41496 \| \| -5.38525 \| \| -5.38107 \| \| -5.37783 \| \| -5.37251 \| \| -5.33856 \| \| -5.33096 \| \| -5.3246 \| \| -5.31304 \| \| -5.31125 \| \| -5.30147 \| \| -5.29187 \| \| -5.27532 \| \| -5.25162 \| \| -5.24517 \| \| -5.2055 \| \| -5.17645 \| \| -5.17143 \| \| -5.1267 \| \| -5.12408 \| \| -5.1051 \| \| -5.10179 \| \| -5.10135 \| \| -5.10118 \| \| -5.10063 \| \| -5.08001 \| \| -5.05615 \| \| -5.05386 \| \| -5.04229 \| \| -5.03332 \| \| -5.03073 \| \| -5.00866 \| \| -4.99604 \| \| -4.98663 \| \| -4.98444 \| \| -4.93229 \| \| -4.91502 \| \| -4.86833 \| \| -4.85596 \| \| -4.8448 \| \| -4.80927 \| \| -4.77494 \| \| -4.76257 \| \| -4.71588 \| \| -4.6915 \| \| -4.63752 \| \| -4.59633 \| \| -4.58677 \| \| -4.55186 \| \| -4.51695 \| \| -4.48204 \| \| -4.45669 \| \| -4.44713 \| \| -4.42075 \| \| -4.38686 \| \| -4.33039 \| \| -4.31883 \| \| -4.31704 \| \| -4.28111 \| \| -4.21129 \| \| -4.1325 \| \| -4.10758 \| \| -4.10732 \| \| -4.10723 \| \| -4.10706 \| \| -4.10697 \| \| -4.0858 \| \| -4.05965 \| \| -4.04809 \| \| -4.03911 \| \| -4.03652 \| \| -4.00183 \| \| -3.9675 \| \| -3.92081 \| \| -3.87412 \| \| -3.86175 \| \| -3.82743 \| \| -3.81506 \| \| -3.78073 \| \| -3.76837 \| \| -3.72167 \| \| -3.69729 \| \| -3.66238 \| \| -3.55765 \| \| -3.52274 \| \| -3.42655 \| \| -3.32462 \| \| -3.31306 \| \| -3.28691 \| \| -3.21709 \| \| -3.0916 \| \| -3.08381 \| \| -3.06544 \| \| -3.05388 \| \| -3.04491 \| \| -3.04232 \| \| -3.00762 \| \| -2.99821 \| \| -2.86755 \| \| -2.82085 \| \| -2.77416 \| \| -2.72747 \| | \| -5.05303 \| \| --- \| \| -0.06751 \| \| 0.261795 \| \| 0.44177 \| \| 1.20001 \| \| 2.467532 \| \| 3.105257 \| \| 3.596187 \| \| 3.67477 \| \| 3.735054 \| \| 3.738868 \| \| 4.223131 \| \| 4.691641 \| \| 4.792644 \| \| 4.901573 \| \| 5.111506 \| \| 5.321438 \| \| 5.636337 \| \| 5.850233 \| \| 5.999881 \| \| 6.164601 \| \| 6.278025 \| \| 6.907823 \| \| 7.755479 \| \| 8.603136 \| \| 9.473586 \| \| 11.73711 \| \| 16.90144 \| \| 17.98129 \| \| 18.49057 \| \| 19.24881 \| \| 21.15406 \| \| 21.22995 \| \| 21.64499 \| \| 21.78385 \| \| 21.78767 \| \| 22.27193 \| \| 22.49747 \| \| 22.84144 \| \| 22.95037 \| \| 23.16031 \| \| 23.37024 \| \| 23.68514 \| \| 23.76499 \| \| 23.89903 \| \| 24.04868 \| \| 24.17718 \| \| 24.32682 \| \| 24.70134 \| \| 25.5847 \| \| 25.80428 \| \| 26.13533 \| \| 26.30004 \| \| 26.65194 \| \| 27.52239 \| \| 29.49017 \| \| 30.1026 \| \| 30.10736 \| \| 30.10895 \| \| 30.11053 \| \| 30.11212 \| \| 30.1137 \| \| 30.13086 \| \| 32.64557 \| \| 34.34089 \| \| 36.03009 \| \| 36.53937 \| \| 37.29761 \| \| 37.49359 \| \| 38.05928 \| \| 38.61196 \| \| 38.91622 \| \| 38.91989 \| \| 39.24863 \| \| 39.31654 \| \| 39.49281 \| \| 39.65133 \| \| 39.8039 \| \| 39.91191 \| \| 39.92507 \| \| 39.96755 \| \| 40.02083 \| \| 40.29584 \| \| 40.31221 \| \| 40.55207 \| \| 40.59316 \| \| 40.68261 \| \| 40.78995 \| \| 41.00361 \| \| 41.0661 \| \| 41.49187 \| \| 41.54565 \| \| 41.59 \| \| 41.66198 \| \| 42.04061 \| \| 42.12163 \| \| 42.21775 \| \| 42.28732 \| \| 42.42137 \| \| 42.54827 \| \| 42.96913 \| \| 43.14183 \| \| 43.68949 \| \| 44.07227 \| \| 44.59651 \| \| 44.78393 \| \| 44.78828 \| \| 44.76099 \| \| 44.71993 \| \| 44.68893 \| \| 44.66043 \| \| 44.63762 \| \| 44.62351 \| \| 44.808 \| \| 45.01682 \| \| 45.12899 \| \| 45.19746 \| \| 45.21255 \| \| 45.38732 \| \| 45.47812 \| \| 45.5454 \| \| 45.56469 \| \| 45.72167 \| \| 45.89603 \| \| 45.98662 \| \| 46.07418 \| \| 46.2237 \| \| 46.28202 \| \| 46.338 \| \| 46.3702 \| \| 46.45832 \| \| 46.50915 \| \| 46.60434 \| \| 46.62177 \| \| 46.64399 \| \| 46.70701 \| \| 46.70137 \| \| 46.70541 \| \| 46.68894 \| \| 46.67921 \| \| 46.69682 \| \| 46.69165 \| \| 46.6907 \| \| 46.68846 \| \| 46.68958 \| \| 46.69656 \| \| 46.69983 \| \| 46.70252 \| \| 46.70714 \| \| 46.71633 \| \| 46.72259 \| \| 46.73919 \| \| 46.74798 \| \| 46.75863 \| \| 46.77847 \| \| 46.77374 \| \| 46.79947 \| \| 46.82012 \| \| 46.85291 \| \| 46.87015 \| \| 46.86011 \| \| 46.89168 \| \| 46.82583 \| \| 46.85218 \| \| 46.74691 \| \| 46.70416 \| \| 46.70062 \| \| 46.74156 \| \| 46.72796 \| \| 46.70994 \| \| 46.64705 \| \| 46.62521 \| \| 46.521 \| \| 46.53741 \| \| 46.31631 \| \| 45.65548 \| \| 45.65652 \| \| 45.66171 \| \| 45.66263 \| \| 45.51254 \| \| 45.32052 \| \| 45.28707 \| \| 45.23013 \| \| 45.15949 \| \| 45.13947 \| \| 44.83899 \| \| 44.75217 \| \| 44.59307 \| \| 44.50898 \| \| 44.05995 \| \| 43.59608 \| \| 43.45696 \| \| 43.08925 \| \| 42.94402 \| \| 42.56359 \| \| 42.54432 \| \| 42.39842 \| \| 42.33586 \| \| 42.20837 \| \| 42.07984 \| \| 41.68718 \| \| 41.50682 \| \| 41.22285 \| \| 40.28344 \| \| 40.14849 \| \| 39.97294 \| \| 39.91584 \| \| 39.87478 \| \| 39.87195 \| \| 39.67864 \| \| 39.49679 \| \| 39.32 \| \| 39.18185 \| \| 39.12743 \| \| 39.04734 \| \| 38.79256 \| \| 38.67673 \| \| 38.35963 \| \| 38.29247 \| \| 38.1458 \| \| 38.04563 \| \| 37.93054 \| \| 37.89772 \| \| 37.8014 \| \| 37.72636 \| \| 37.25296 \| \| 37.19277 \| \| 36.6034 \| \| 36.4983 \| \| 36.06831 \| \| 36.03638 \| \| 35.86843 \| \| 35.42061 \| \| 35.27819 \| \| 34.88489 \| \| 34.20659 \| \| 32.78045 \| \| 32.63906 \| \| 32.32306 \| \| 32.3333 \| \| 32.33609 \| \| 32.34647 \| \| 32.35063 \| \| 32.3418 \| \| 31.92092 \| \| 31.17417 \| \| 30.99942 \| \| 30.9588 \| \| 30.25748 \| \| 30.07403 \| \| 29.56766 \| \| 28.59425 \| \| 27.33766 \| \| 26.87544 \| \| 26.59362 \| \| 26.32736 \| \| 25.57756 \| \| 25.57182 \| \| 25.2936 \| \| 24.94547 \| \| 23.97459 \| \| 23.11901 \| \| 22.8115 \| \| 21.35861 \| \| 21.11753 \| \| 20.76677 \| \| 20.63489 \| \| 20.536 \| \| 20.51801 \| \| 20.14785 \| \| 19.7966 \| \| 19.4498 \| \| 19.1742 \| \| 19.05117 \| \| 18.88208 \| \| 18.70214 \| \| 18.36991 \| \| 18.13527 \| \| 17.54124 \| \| 17.40313 \| \| 17.11508 \| \| 16.92245 \| \| 16.70435 \| \| 16.30821 \| \| 14.26693 \| \| 14.05244 \| \| 13.61718 \| \| 13.46785 \| \| 13.24511 \| \| 13.18846 \| \| 12.88112 \| \| 12.57501 \| \| 12.05435 \| \| 11.77386 \| \| 9.830466 \| \| 7.566877 \| \| 7.240439 \| \| 6.391243 \| \| 6.349991 \| \| 6.319659 \| \| 6.290673 \| \| 6.262288 \| \| 6.206511 \| \| 5.506804 \| \| 4.189018 \| \| 3.865235 \| \| 3.764954 \| \| 2.811367 \| \| 2.510002 \| \| 1.265567 \| \| -2.16413 \| \| -2.66198 \| \| -3.99643 \| \| -4.52146 \| \| -4.73571 \| \| -5.93611 \| \| -6.01243 \| \| -6.55623 \| \| -7.23308 \| \| -8.5732 \| \| -9.08874 \| \| -9.76207 \| \| -10.7903 \| \| -11.4164 \| \| -12.4168 \| \| -14.0615 \| \| -14.7763 \| \| -15.6668 \| \| -15.9106 \| \| -16.0054 \| \| -17.3534 \| \| -18.0395 \| \| -18.5833 \| \| -18.8439 \| \| -19.1911 \| \| -19.5634 \| \| -20.6145 \| \| -21.7453 \| \| -22.0128 \| \| -22.5445 \| \| -22.8872 \| \| -23.2754 \| \| -23.9943 \| \| -25.6194 \| \| -25.8354 \| \| -25.9877 \| \| -26.251 \| \| -28.1457 \| \| -28.5415 \| \| -28.8568 \| \| -29.4694 \| \| -29.4994 \| \| -30.0153 \| \| -30.5163 \| \| -31.4383 \| \| -32.7982 \| \| -33.0937 \| \| -35.4607 \| \| -37.1907 \| \| -37.4078 \| \| -40.172 \| \| -40.2433 \| \| -41.3828 \| \| -41.52 \| \| -41.4558 \| \| -41.3707 \| \| -41.2922 \| \| -42.5561 \| \| -44.0385 \| \| -44.0525 \| \| -44.6893 \| \| -45.1486 \| \| -45.1732 \| \| -46.5561 \| \| -47.2799 \| \| -47.7781 \| \| -47.7621 \| \| -51.4552 \| \| -52.6786 \| \| -55.9854 \| \| -56.8613 \| \| -57.6518 \| \| -60.1682 \| \| -62.5992 \| \| -63.475 \| \| -66.7819 \| \| -68.5087 \| \| -72.3318 \| \| -75.249 \| \| -75.926 \| \| -78.3984 \| \| -80.8708 \| \| -83.3433 \| \| -85.1387 \| \| -85.8157 \| \| -87.6834 \| \| -90.0836 \| \| -94.0829 \| \| -94.9019 \| \| -95.0284 \| \| -97.5731 \| \| -102.518 \| \| -108.098 \| \| -109.863 \| \| -109.882 \| \| -109.888 \| \| -109.9 \| \| -109.906 \| \| -111.405 \| \| -113.258 \| \| -114.077 \| \| -114.712 \| \| -114.896 \| \| -117.353 \| \| -119.784 \| \| -123.09 \| \| -126.397 \| \| -127.273 \| \| -129.704 \| \| -130.58 \| \| -133.011 \| \| -133.887 \| \| -137.194 \| \| -138.921 \| \| -141.393 \| \| -148.81 \| \| -151.283 \| \| -158.095 \| \| -165.314 \| \| -166.133 \| \| -167.985 \| \| -172.93 \| \| -181.817 \| \| -182.369 \| \| -183.669 \| \| -184.488 \| \| -185.124 \| \| -185.307 \| \| -187.764 \| \| -188.431 \| \| -197.685 \| \| -200.992 \| \| -204.299 \| \| -207.606 \| | \| -13.215 \| \| --- \| \| -13.0216 \| \| -12.9017 \| \| -12.8282 \| \| -12.5097 \| \| -12.2963 \| \| -12.2384 \| \| -12.2196 \| \| -12.1297 \| \| -12.0262 \| \| -11.9363 \| \| -11.9296 \| \| -11.8516 \| \| -11.8329 \| \| -11.7549 \| \| -11.7429 \| \| -11.6958 \| \| -11.677 \| \| -11.6582 \| \| -11.6212 \| \| -11.5803 \| \| -11.5615 \| \| -11.4836 \| \| -11.4245 \| \| -11.4057 \| \| -11.3869 \| \| -11.3499 \| \| -11.325 \| \| -11.309 \| \| -11.2902 \| \| -11.2392 \| \| -11.2222 \| \| -11.2123 \| \| -11.211 \| \| -11.1532 \| \| -11.1344 \| \| -11.1156 \| \| -11.0786 \| \| -11.0537 \| \| -11.0288 \| \| -11.0189 \| \| -10.9509 \| \| -10.941 \| \| -10.8819 \| \| -10.8631 \| \| -10.8443 \| \| -10.8072 \| \| -10.7824 \| \| -10.7664 \| \| -10.7575 \| \| -10.7476 \| \| -10.7326 \| \| -10.6796 \| \| -10.6697 \| \| -10.5918 \| \| -10.573 \| \| -10.5359 \| \| -10.5217 \| \| -10.5111 \| \| -10.4951 \| \| -10.4613 \| \| -10.4365 \| \| -10.4083 \| \| -10.3984 \| \| -10.3639 \| \| -10.2791 \| \| -10.2504 \| \| -10.2398 \| \| -10.2149 \| \| -10.205 \| \| -10.19 \| \| -10.1651 \| \| -10.137 \| \| -10.0926 \| \| -9.99331 \| \| -9.9791 \| \| -9.96196 \| \| -9.94357 \| \| -9.9187 \| \| -9.89383 \| \| -9.86553 \| \| -9.82124 \| \| -9.69713 \| \| -9.69065 \| \| -9.67226 \| \| -9.67187 \| \| -9.66338 \| \| -9.64739 \| \| -9.62252 \| \| -9.59434 \| \| -9.59422 \| \| -9.57544 \| \| -9.52709 \| \| -9.47874 \| \| -9.47848 \| \| -9.43648 \| \| -9.41933 \| \| -9.40056 \| \| -9.39601 \| \| -9.39207 \| \| -9.38205 \| \| -9.38178 \| \| -9.35248 \| \| -9.3337 \| \| -9.32291 \| \| -9.30413 \| \| -9.30386 \| \| -9.28535 \| \| -9.27862 \| \| -9.25578 \| \| -9.20743 \| \| -9.20716 \| \| -9.1939 \| \| -9.18541 \| \| -9.17315 \| \| -9.16517 \| \| -9.15908 \| \| -9.14802 \| \| -9.12925 \| \| -9.12076 \| \| -9.11047 \| \| -9.10477 \| \| -9.08116 \| \| -9.07989 \| \| -9.07672 \| \| -9.06239 \| \| -9.05159 \| \| -9.03282 \| \| -9.03255 \| \| -9.01404 \| \| -9.00731 \| \| -8.98447 \| \| -8.96289 \| \| -8.93612 \| \| -8.93585 \| \| -8.91022 \| \| -8.90184 \| \| -8.88777 \| \| -8.87671 \| \| -8.85793 \| \| -8.84944 \| \| -8.83916 \| \| -8.81379 \| \| -8.80985 \| \| -8.80858 \| \| -8.80541 \| \| -8.7615 \| \| -8.76124 \| \| -8.74273 \| \| -8.73599 \| \| -8.71316 \| \| -8.69158 \| \| -8.67444 \| \| -8.66481 \| \| -8.66454 \| \| -8.63891 \| \| -8.63053 \| \| -8.61646 \| \| -8.6054 \| \| -8.58662 \| \| -8.56785 \| \| -8.55086 \| \| -8.54616 \| \| -8.54248 \| \| -8.53854 \| \| -8.5341 \| \| -8.51976 \| \| -8.49019 \| \| -8.48993 \| \| -8.44184 \| \| -8.42027 \| \| -8.40313 \| \| -8.37597 \| \| -8.3676 \| \| -8.35922 \| \| -8.3549 \| \| -8.34515 \| \| -8.33409 \| \| -8.31531 \| \| -8.30682 \| \| -8.29653 \| \| -8.27954 \| \| -8.27117 \| \| -8.26723 \| \| -8.26279 \| \| -8.24845 \| \| -8.24527 \| \| -8.21888 \| \| -8.21861 \| \| -8.2001 \| \| -8.17053 \| \| -8.14896 \| \| -8.14884 \| \| -8.13182 \| \| -8.12219 \| \| -8.10016 \| \| -8.09628 \| \| -8.08999 \| \| -8.08791 \| \| -8.08359 \| \| -8.07384 \| \| -8.044 \| \| -8.03539 \| \| -8.02522 \| \| -7.99985 \| \| -7.99148 \| \| -7.97395 \| \| -7.97176 \| \| -7.9473 \| \| -7.92879 \| \| -7.89922 \| \| -7.87765 \| \| -7.87752 \| \| -7.87047 \| \| -7.8605 \| \| -7.85087 \| \| -7.82497 \| \| -7.8166 \| \| -7.81609 \| \| -7.81228 \| \| -7.76407 \| \| -7.75391 \| \| -7.73692 \| \| -7.72854 \| \| -7.71966 \| \| -7.70583 \| \| -7.70264 \| \| -7.65748 \| \| -7.60621 \| \| -7.58919 \| \| -7.56204 \| \| -7.55366 \| \| -7.54528 \| \| -7.54478 \| \| -7.54096 \| \| -7.49276 \| \| -7.46561 \| \| -7.46092 \| \| -7.45723 \| \| -7.44885 \| \| -7.44835 \| \| -7.43133 \| \| -7.3349 \| \| -7.31788 \| \| -7.29073 \| \| -7.29049 \| \| -7.28235 \| \| -7.27347 \| \| -7.26965 \| \| -7.22145 \| \| -7.1943 \| \| -7.18592 \| \| -7.17704 \| \| -7.16002 \| \| -7.06359 \| \| -7.04657 \| \| -7.01941 \| \| -7.01918 \| \| -7.00474 \| \| -7.00216 \| \| -6.99834 \| \| -6.975 \| \| -6.95775 \| \| -6.90573 \| \| -6.89145 \| \| -6.88871 \| \| -6.79228 \| \| -6.78522 \| \| -6.74787 \| \| -6.73085 \| \| -6.72703 \| \| -6.68644 \| \| -6.67525 \| \| -6.67279 \| \| -6.63442 \| \| -6.6174 \| \| -6.57298 \| \| -6.52097 \| \| -6.45953 \| \| -6.41512 \| \| -6.40148 \| \| -6.36311 \| \| -6.30167 \| \| -6.20524 \| \| -6.18822 \| \| -6.14381 \| \| -6.09179 \| \| -6.04156 \| \| -6.03036 \| \| -5.89651 \| \| -5.85885 \| \| -5.79982 \| \| -5.77025 \| \| -5.75905 \| \| -5.73027 \| \| -5.70312 \| \| -5.66262 \| \| -5.64716 \| \| -5.63866 \| \| -5.6252 \| \| -5.60642 \| \| -5.59 \| \| -5.58754 \| \| -5.56404 \| \| -5.55886 \| \| -5.55503 \| \| -5.52386 \| \| -5.49893 \| \| -5.45896 \| \| -5.43181 \| \| -5.3978 \| \| -5.38422 \| \| -5.37584 \| \| -5.35389 \| \| -5.33511 \| \| -5.31623 \| \| -5.31412 \| \| -5.29272 \| \| -5.28755 \| \| -5.25255 \| \| -5.25241 \| \| -5.24458 \| \| -5.22762 \| \| -5.18765 \| \| -5.1605 \| \| -5.12129 \| \| -5.11291 \| \| -5.10771 \| \| -5.10453 \| \| -5.08258 \| \| -5.0638 \| \| -5.04492 \| \| -5.02141 \| \| -4.98124 \| \| -4.97327 \| \| -4.96862 \| \| -4.95631 \| \| -4.91634 \| \| -4.88918 \| \| -4.85835 \| \| -4.85517 \| \| -4.84997 \| \| -4.8364 \| \| -4.83322 \| \| -4.81127 \| \| -4.79249 \| \| -4.7501 \| \| -4.74492 \| \| -4.70993 \| \| -4.70196 \| \| -4.69731 \| \| -4.685 \| \| -4.65714 \| \| -4.64503 \| \| -4.61787 \| \| -4.60899 \| \| -4.59301 \| \| -4.58386 \| \| -4.57866 \| \| -4.57301 \| \| -4.57029 \| \| -4.56509 \| \| -4.55341 \| \| -4.53995 \| \| -4.53945 \| \| -4.52118 \| \| -4.50019 \| \| -4.47361 \| \| -4.46978 \| \| -4.43861 \| \| -4.43065 \| \| -4.426 \| \| -4.41369 \| \| -4.40324 \| \| -4.37372 \| \| -4.34656 \| \| -4.33768 \| \| -4.31573 \| \| -4.31255 \| \| -4.30735 \| \| -4.29897 \| \| -4.29646 \| \| -4.26864 \| \| -4.26814 \| \| -4.24987 \| \| -4.22888 \| \| -4.17195 \| \| -4.1673 \| \| -4.16716 \| \| -4.15933 \| \| -4.1024 \| \| -4.07525 \| \| -4.06637 \| \| -4.02766 \| \| -3.97855 \| \| -3.95756 \| \| -3.89599 \| \| -3.88802 \| \| -3.88338 \| \| -3.8432 \| \| -3.79506 \| \| -3.7731 \| \| -3.76993 \| \| -3.76473 \| \| -3.72552 \| \| -3.68625 \| \| -3.61671 \| \| -3.61207 \| \| -3.57189 \| \| -3.52375 \| \| -3.50777 \| \| -3.50179 \| \| -3.49342 \| \| -3.48776 \| \| -3.47984 \| \| -3.34075 \| \| -3.31799 \| \| -3.30058 \| \| -3.29634 \| \| -3.23048 \| \| -3.21121 \| \| -3.18289 \| \| -3.08371 \| \| -2.91158 \| \| -2.8124 \| \| -2.80598 \| \| -2.54109 \| \| -2.51795 \| \| -2.48241 \| \| -2.26977 \| \| -2.22287 \| \| -1.99846 \| \| -1.90448 \| \| -1.72715 \| \| -1.55965 \| \| -1.4327 \| \| -1.35016 \| \| -1.13763 \| \| -0.91103 \| \| -0.4744 \| \| 2.422113 \| \| 3.50736 \| \| 5.514313 \| \| 6.59956 \| | \| -232.599 \| \| --- \| \| -221.29 \| \| -214.283 \| \| -209.981 \| \| -191.359 \| \| -178.877 \| \| -175.494 \| \| -174.396 \| \| -169.138 \| \| -163.087 \| \| -157.829 \| \| -157.432 \| \| -152.876 \| \| -151.778 \| \| -147.221 \| \| -146.52 \| \| -143.763 \| \| -142.665 \| \| -141.567 \| \| -139.4 \| \| -137.01 \| \| -135.912 \| \| -131.356 \| \| -127.897 \| \| -126.799 \| \| -125.701 \| \| -123.534 \| \| -122.08 \| \| -121.145 \| \| -120.047 \| \| -117.061 \| \| -116.069 \| \| -115.49 \| \| -115.415 \| \| -112.032 \| \| -110.934 \| \| -109.836 \| \| -107.669 \| \| -106.215 \| \| -104.76 \| \| -104.181 \| \| -100.204 \| \| -99.625 \| \| -96.1665 \| \| -95.0685 \| \| -93.9705 \| \| -91.8035 \| \| -90.3491 \| \| -89.414 \| \| -88.8948 \| \| -88.316 \| \| -87.4404 \| \| -84.3383 \| \| -83.7596 \| \| -79.2031 \| \| -78.1051 \| \| -75.9381 \| \| -75.1071 \| \| -74.4837 \| \| -73.5486 \| \| -71.575 \| \| -70.1207 \| \| -68.4729 \| \| -67.8942 \| \| -65.8759 \| \| -60.9219 \| \| -59.2417 \| \| -58.6183 \| \| -57.1688 \| \| -56.5948 \| \| -55.7254 \| \| -54.2869 \| \| -52.6684 \| \| -50.1189 \| \| -44.4268 \| \| -43.6434 \| \| -42.6886 \| \| -41.665 \| \| -40.2783 \| \| -38.902 \| \| -37.336 \| \| -34.8762 \| \| -27.9146 \| \| -27.605 \| \| -26.6204 \| \| -26.6489 \| \| -26.2253 \| \| -25.3777 \| \| -24.0457 \| \| -22.5331 \| \| -22.5777 \| \| -21.5892 \| \| -18.9892 \| \| -16.4137 \| \| -16.4805 \| \| -14.2702 \| \| -13.4156 \| \| -12.4622 \| \| -12.2819 \| \| -12.1172 \| \| -11.6174 \| \| -11.6318 \| \| -10.1111 \| \| -9.16035 \| \| -8.63018 \| \| -7.68634 \| \| -7.71301 \| \| -6.79225 \| \| -6.49172 \| \| -5.3602 \| \| -2.91712 \| \| -2.94449 \| \| -2.30166 \| \| -1.9059 \| \| -1.32114 \| \| -0.94924 \| \| -0.66548 \| \| -0.12986 \| \| 0.787334 \| \| 1.188605 \| \| 1.677283 \| \| 1.946642 \| \| 3.080499 \| \| 3.124756 \| \| 3.255278 \| \| 3.919925 \| \| 4.418664 \| \| 5.291773 \| \| 5.296431 \| \| 6.149979 \| \| 6.460419 \| \| 7.498409 \| \| 8.470156 \| \| 9.670149 \| \| 9.678971 \| \| 10.81944 \| \| 11.19376 \| \| 11.81286 \| \| 12.31504 \| \| 13.14107 \| \| 13.51515 \| \| 13.9546 \| \| 15.02197 \| \| 15.17647 \| \| 15.21458 \| \| 15.34248 \| \| 17.14326 \| \| 17.14776 \| \| 17.89948 \| \| 18.1698 \| \| 19.06739 \| \| 19.91343 \| \| 20.59219 \| \| 20.96765 \| \| 20.98159 \| \| 21.99181 \| \| 22.33092 \| \| 22.8845 \| \| 23.3311 \| \| 24.06703 \| \| 24.77869 \| \| 25.40913 \| \| 25.57819 \| \| 25.70655 \| \| 25.83911 \| \| 25.97074 \| \| 26.46819 \| \| 27.50305 \| \| 27.495 \| \| 29.13069 \| \| 29.83631 \| \| 30.39603 \| \| 31.28326 \| \| 31.53783 \| \| 31.78732 \| \| 31.88643 \| \| 32.14863 \| \| 32.45106 \| \| 33.01803 \| \| 33.24909 \| \| 33.54954 \| \| 34.05817 \| \| 34.29995 \| \| 34.40753 \| \| 34.51607 \| \| 34.91484 \| \| 34.99666 \| \| 35.69694 \| \| 35.67289 \| \| 36.13328 \| \| 36.85089 \| \| 37.34546 \| \| 37.31582 \| \| 37.69704 \| \| 37.89392 \| \| 38.37325 \| \| 38.42556 \| \| 38.54052 \| \| 38.58159 \| \| 38.66487 \| \| 38.85745 \| \| 39.45154 \| \| 39.60895 \| \| 39.79678 \| \| 40.26577 \| \| 40.41147 \| \| 40.72955 \| \| 40.77781 \| \| 41.20279 \| \| 41.52615 \| \| 42.01037 \| \| 42.34599 \| \| 42.36326 \| \| 42.50169 \| \| 42.67476 \| \| 42.84643 \| \| 43.22346 \| \| 43.37437 \| \| 43.4326 \| \| 43.52617 \| \| 44.07095 \| \| 44.2218 \| \| 44.43547 \| \| 44.57233 \| \| 44.71482 \| \| 44.90256 \| \| 45.01424 \| \| 45.40061 \| \| 45.79899 \| \| 45.98539 \| \| 46.21483 \| \| 46.35313 \| \| 46.48497 \| \| 46.60341 \| \| 46.72025 \| \| 46.95613 \| \| 47.11194 \| \| 47.19644 \| \| 47.28334 \| \| 47.37736 \| \| 47.45747 \| \| 47.56571 \| \| 47.74621 \| \| 47.84573 \| \| 47.94453 \| \| 48.02022 \| \| 48.09691 \| \| 48.17288 \| \| 48.25824 \| \| 48.30724 \| \| 48.36314 \| \| 48.43266 \| \| 48.50375 \| \| 48.55998 \| \| 48.4666 \| \| 48.49752 \| \| 48.50296 \| \| 48.56383 \| \| 48.59022 \| \| 48.65747 \| \| 48.73179 \| \| 48.73699 \| \| 48.75785 \| \| 48.64382 \| \| 48.66741 \| \| 48.73969 \| \| 48.41567 \| \| 48.4723 \| \| 48.37719 \| \| 48.36932 \| \| 48.42034 \| \| 48.27819 \| \| 48.2836 \| \| 48.32978 \| \| 48.16505 \| \| 48.12591 \| \| 47.91916 \| \| 47.65282 \| \| 47.32363 \| \| 47.08959 \| \| 47.05058 \| \| 46.84013 \| \| 46.4578 \| \| 45.80901 \| \| 45.7105 \| \| 45.41323 \| \| 45.04459 \| \| 44.68035 \| \| 44.61046 \| \| 43.57272 \| \| 43.27645 \| \| 42.80882 \| \| 42.57085 \| \| 42.47918 \| \| 42.2445 \| \| 42.02386 \| \| 41.67779 \| \| 41.54077 \| \| 41.46284 \| \| 41.36582 \| \| 41.22281 \| \| 41.09598 \| \| 41.08381 \| \| 40.87302 \| \| 40.82325 \| \| 40.78242 \| \| 40.50389 \| \| 40.28582 \| \| 39.92651 \| \| 39.68034 \| \| 39.3652 \| \| 39.24059 \| \| 39.16279 \| \| 38.94996 \| \| 38.77005 \| \| 38.58268 \| \| 38.55171 \| \| 38.33492 \| \| 38.27203 \| \| 37.92465 \| \| 37.91227 \| \| 37.82752 \| \| 37.65427 \| \| 37.25427 \| \| 36.97829 \| \| 36.57716 \| \| 36.48354 \| \| 36.41881 \| \| 36.37832 \| \| 36.14193 \| \| 35.9386 \| \| 35.73411 \| \| 35.47565 \| \| 35.02892 \| \| 34.92905 \| \| 34.87038 \| \| 34.72701 \| \| 34.27244 \| \| 33.9568 \| \| 33.59443 \| \| 33.55089 \| \| 33.48483 \| \| 33.3199 \| \| 33.28447 \| \| 33.01844 \| \| 32.78988 \| \| 32.26935 \| \| 32.20602 \| \| 31.75994 \| \| 31.65728 \| \| 31.60112 \| \| 31.44009 \| \| 31.07663 \| \| 30.91941 \| \| 30.56358 \| \| 30.44869 \| \| 30.23628 \| \| 30.10424 \| \| 30.02762 \| \| 29.94511 \| \| 29.89411 \| \| 29.81116 \| \| 29.63923 \| \| 29.43922 \| \| 29.41897 \| \| 29.14615 \| \| 28.83179 \| \| 28.4408 \| \| 28.37592 \| \| 27.92423 \| \| 27.81719 \| \| 27.76644 \| \| 27.60049 \| \| 27.49229 \| \| 27.08504 \| \| 26.71272 \| \| 26.61625 \| \| 26.26563 \| \| 26.21489 \| \| 26.13184 \| \| 25.99805 \| \| 25.95792 \| \| 25.51364 \| \| 25.50561 \| \| 25.21376 \| \| 24.87854 \| \| 23.96934 \| \| 23.89517 \| \| 23.89289 \| \| 23.76791 \| \| 22.85871 \| \| 22.42504 \| \| 22.28322 \| \| 21.66502 \| \| 20.88073 \| \| 20.54551 \| \| 19.56214 \| \| 19.43488 \| \| 19.36071 \| \| 18.71908 \| \| 17.95019 \| \| 17.59957 \| \| 17.54883 \| \| 17.46578 \| \| 16.83956 \| \| 16.21248 \| \| 15.10185 \| \| 15.02768 \| \| 14.38606 \| \| 13.61717 \| \| 13.36198 \| \| 13.26654 \| \| 13.13276 \| \| 13.04244 \| \| 12.91592 \| \| 10.69466 \| \| 10.33108 \| \| 10.05303 \| \| 9.985383 \| \| 8.933516 \| \| 8.625807 \| \| 8.173505 \| \| 6.589467 \| \| 3.840478 \| \| 2.256439 \| \| 2.153928 \| \| -2.07659 \| \| -2.44613 \| \| -3.0137 \| \| -6.40962 \| \| -7.15864 \| \| -10.7426 \| \| -12.2435 \| \| -15.0757 \| \| -17.7508 \| \| -19.7782 \| \| -21.0964 \| \| -24.4908 \| \| -28.1097 \| \| -35.0829 \| \| -81.3422 \| \| -98.6743 \| \| -130.727 \| \| -148.059 \| |
