# Supplementary material for: Non-hypothetical projection pursuit regression for the prediction of hydration heat of Portland-cement-based cementitious system
Source: Heliyon. 2023 Aug 28;9(9):e19471. doi: 10.1016/j.heliyon.2023.e19471 (PMC10480638; doi:10.1016/j.heliyon.2023.e19471)
Supplement: Multimedia component 5 [file mmc5.docx]

The optimization process is as follows:

Step 1: The *N*-dimensional dependent variable is synthesized into a one-dimensional form based on the given weight coefficients **$\mu_{k}$** of the ridge function, which is the fitting target of the *k*th ridge function.

Step 2: The initial linear coefficient******and increment******of the *k*th ridge function are set. The determination of the linear coefficients******first requires an assumption of the finite difference:$\frac{\Delta G}{\Delta T}(m)=1$. The *T* is the horizontal axis of the *k*th ridge function. The matrix for the finite differences$\left(\frac{\Delta G}{\Delta\eta_{mk}}\right)_{M\times N}$ is then calculated, and finally the error *S* is minimized using an iterative optimization method to find the optimal value of the linear coefficient******. The matrix for the finite difference$\left(\frac{\Delta G}{\Delta\eta_{mk}}\right)_{M\times N}$and the error *S* are calculated as follows:

$\frac{\Delta G}{\Delta\eta_{mk}}(m)=\frac{\Delta G}{\Delta T}(m)\times\frac{\Delta T}{\Delta\eta_{mk}}(m)$ (3)

$S=\sum_{m}^{M} W(m)\times\left[YZ(m)-\sum_{m}^{M} \eta_{mk}\times\frac{\Delta G}{\eta_{mk}}(m)^{2}\right]$ (4)

Step 3: The linear coefficient ***$\eta_{mk}$*** of the *k*th ridge function is calculated, and the modulus is set as 1.

Step 4: The horizontal axis *T*(*j*) of the *k*th ridge function is calculated.

Step 5: The super-filter method smooths the *YZ*-*T* data, which can be adjusted by smoothing coefficient (*Span*). Moreover, the *Span* decides the sensitivity of model based on the precision of data set. The lower the value of Span, the more the sensitivity it is. Besides, the numerical solution of ridge function *G*(*m*) is obtained.

Step 6: The squared mean of the residual error *S* of the *k*th ridge function is calculated. If *S*=1, the optimization effect is not apparent. When the number of iterations is greater than 20, the optimization process of the linear coefficient ******is finished, and then the parameter**is optimized in the second layer. Otherwise, the Newton gradient method or bisection method is used to update ***$\Delta\eta_{mk}$***, and then the process returns to step 3.

Within the optimization layer of**,**is calculated according to the value of the *k*th ridge function. The total loss *L*_2_ of the response quantity is calculated according to Equation (2). If the optimization effect of *M*_2_ is not apparent or the number of iterations is greater than 20, the optimization process of the *k*th ridge function is ended, and the residual matrix *R* is calculated to prepare the fit of the next ridge function; otherwise, the optimization process is repeated. If the total loss *M*_2_ = 0 or its decay is not apparent, the terms of the ridge function are no longer increased, and the parameter optimization process is ended.
